# Orangutans and chimpanzees show evidence of inferring when a hidden breadstick is intact or broken

**DOI:** 10.1038/s41598-026-38796-x

**Published:** 2026-02-27

**Authors:** Michèle N. Schubiger, Claudia Fichtel, Nicholas J. Mulcahy

**Affiliations:** 1 Behavioural Ecology & Sociobiology Unit, German Primate Center, Göttingen, Germany; 2 Leibniz Science Campus ‘Primate Cognition’, Göttingen, Germany; 3 Department of Evolutionary Anthropology, University of Zurich, Zürich, Switzerland; 4 World Ape Fund, London, UK

**Keywords:** Inferential reasoning, Primate cognition, Inference by exclusion, 2-cups 1-item task, Orangutans, Chimpanzees, Biological techniques, Evolution, Neuroscience, Psychology, Psychology, Zoology

## Abstract

Evidence suggesting non-human primates infer the hidden functional properties of tools is equivocal, possibly because subjects had to reason about two factors: the tools’ functional properties and the out-of-reach reward. We designed a novel inference task with a simplified set-up in which subjects only had to reason about one factor. Subjects could choose between a breadstick that was intact or broken. If subjects chose the intact breadstick they could eat all of it, whereas choosing the broken breadstick would mean they could only eat less than half or, in some cases, none at all. Covers were placed over the breadsticks’ middle sections that hid their broken or intact properties but allowed their ends to protrude. The breadsticks now looked identical but it was inferable which one was intact by watching the experimenter move one end of each breadstick, which moved both ends of the intact breadstick but not the broken breadstick’s unattached end. We found that only one of the 12 subjects chose the intact breadstick significantly above chance. In a series of follow-up experiments, we investigated if procedural modifications would improve the subjects’ performance. Although some experiments resulted in more subjects being successful, learning during the test procedures could not be excluded. Therefore, we tested subjects with novel types of breadstick configurations and most subjects passed these transfer tests suggesting that orangutans and chimpanzees show some evidence of inferring the intact and broken properties of breadsticks in this task.

**Supplementary Information:**

The online version contains supplementary material available at 10.1038/s41598-026-38796-x.

## Introduction

Being able to use perceivable information to infer events one cannot directly observe is a central element of human cognition. Engaging in such inferential reasoning allows an individual to optimally deal with and solve problems in its physical and social environment when some relevant information is missing. In doing so, human individuals mentally represent and relate the events they can directly perceive and those events they can only infer or imagine^1^. An intriguing question is if non-human animals (hereafter animals) are also capable of inferring unobservable events in a similar way. However, it is currently debatable if, and to what extent, this ability is present in animals.

One predominant experimental paradigm to test the inferential abilities of animals is the 2-cup 1-item task (hereafter cup task) that tests the ability for inferential reasoning by exclusion. Devised by Grether & Maslow^2^, and later modified by Call^3^, the task involves showing subjects two empty opaque cups followed by an experimenter surreptitiously hiding food under one of the cups. Subjects do not know which cup is empty until the experimenter briefly lifts the empty cup to show that the reward is not hidden underneath. Subjects are then allowed to choose just one of the cups to find the reward. The rationale of this experiment is that an individual capable of inferential reasoning by exclusion will mentally represent that the cups are related in space and time and successfully use the visual cue to reason that because the food is not under the empty cup (A) it must be under the other cup (B), if not A, then B. Several species have demonstrated success in the cup task (and with modifications to the task), such as great apes^3–5^; capuchin monkeys (*Cebus apella*)^6,7^; olive baboons (*Papio hamadryas anubis*)^8^; lion-tailed macaques (*Macaca silenus*)^9^; dogs (*Canis familiaris*)^10^; pigs (*Sus scrofa domestica*)^11^; goats (*Ovis orientalis aries*)^12^; African grey parrots (*Psittacus erithacus*)^13,14^.

However, an alternative non-inferential explanation of subjects passing the cup task is that subjects use a simple rule of avoiding the empty cup and therefore they choose the baited cup without having any expectation that the reward is hidden inside^7,15^. Recently Call^16^ found little evidence to support the empty cup hypothesis by using a 3-cup 1-item task to test the inferential abilities of great apes (*Gorilla gorilla*,* Pan paniscus*,* Pan troglodytes*,* Pongo abelii*). In one experiment, subjects were shown that three opaque cups were empty before two were hidden behind a barrier with the third cup remaining visible to the subjects. The experimenter then hid food under one of the two cups that were behind the barrier. After removing the barrier, the experimenter then either revealed to the subject the contents of the baited cup before removing it along with the reward, or he showed that one cup was empty before removing this empty cup. Subjects were then faced with the task of selecting between two cups with unknown contents: one that had been behind the barrier and one that had always been visible. If subjects had no expectation about the location of the reward and were simply avoiding the empty cup, they should have no preference for choosing the cup that had been behind the barrier. However, based on object permanence, subjects observing the reward going behind the barrier should reason that the experimenter has hidden the reward in one of the two cups that are also behind the barrier. When subjects see the empty cup being removed, they should therefore prefer to choose the cup that had been behind the barrier as this is expected to contain the reward. But when they see the reward and its cup being removed, they should decrease their preference for the cup behind the barrier because they expect it to be empty. This is exactly what the results revealed suggesting that subjects were not basing their choices on avoiding the empty cup. However, Call^16^ acknowledged that it is conceivable that subjects may have used a more sophisticated form of ‘avoid the empty cup’ strategy.

It is therefore useful to use an inferential reasoning paradigm that controls for such a strategy. One such paradigm was used by Mulcahy & Schubiger^17^ who investigated if orangutans (*Pongo abelii*) could infer the hidden functional properties of wooden sticks. The study was based on Mulcahy et al.’s, (2013) study that showed that orangutans understand the observable functional properties of natural tools (sticks made from tree branches). Mulcahy et al.^18^ presented orangutans with two sticks that each skewered a grape that was out of the subject’s reach. An intact tool was connected to the reward and therefore functional allowing the subject to pull it to retrieve the skewered grape. The second tool, however, was broken and therefore not fully connected to the reward meaning it could not be used to retrieve the grape. In a series of experiments the orangutans consistently demonstrated that they understood the functional properties of the tools by consistently pulling in the intact tool to retrieve the reward. These results were consistent with other studies that had also shown evidence of various species understanding similar connectivity tasks using material such as string, cloth and less ecologically valid tools such as rakes (for a review see^19^).

Mulcahy & Schubiger’s^17^ inference study used a modified version of the skewered grape task and this had the potential to conclusively rule out the avoid-empty-cup hypothesis of Call’s^3^ study because subjects were never presented with an empty “cup”. Instead, subjects were presented with two tools skewering a grape, and the tools’ functional properties were concealed using boxes rather than cups, which allowed the ends of the tools to protrude from the boxes. In one experiment, subjects were presented with the broken tool’s protruding ends clearly misaligned to indirectly indicate it was broken compared to the functional tool that had its protruding ends aligned by virtue of its intact state. All subjects significantly chose the intact tool over the broken one indicating they could infer tool functionality. However, subsequent control conditions suggested that subjects may have based their choices on a non-inferential rule about the configuration of the broken tools.

If great apes can make correct inferences about the food location in the cup task, it is puzzling why they cannot make the same inferences about the location of tool functionality that uses a similar set-up to the cup task, especially when great apes can correctly search for hidden tool functionality in other (non-inferential) tasks^20,21^. It is possible, therefore, that the positive results in the cup task can be explained by the avoid empty cup strategy. Alternatively, great apes may have shown evidence of inferential reasoning in the cup task but found it difficult to demonstrate the same ability in the tools task. Maybe the task demands of the tools task were too high, or the subjects were distracted because they had to reason about two factors simultaneously: the functional properties of the tools and the out-of-reach reward.

In the current study we aimed to overcome these potential limitations by using a novel inferential reasoning task with breadsticks that was similar to Mulcahy & Schubiger’s inference task but less demanding in that subjects were only required to represent and reason about one factor. Breadsticks were presented in a way that hid their broken or intact properties (hereafter properties) but these properties could be inferred based on a range of indirect visual cues we provided. The task therefore required integrating perceptual information with causal understanding to draw conclusions about partially hidden objects. Specifically, inferring whether a breadstick was whole or broken from differences in the movement of its visible ends goes beyond simple visual tracking and depends on understanding how the parts of an object are causally connected. In addition, if we found evidence that subjects could infer the breadsticks’ properties such findings could not be explained by the empty cup hypothesis because no container was empty during testing. We conducted a series of 10 experiments, each of which we will sequentially describe and discuss in this paper.

## Familiarisation and establishing if subjects had a preference for an intact breadstick

Before test trials, we familiarised subjects with the basic test setup and procedure by presenting them with two breadsticks: one intact and one broken. Subjects could consume the full breadstick if they chose it, but they could only consume half of the broken breadstick if they chose it. The procedure involved subjects observing each breadstick being covered by an opaque open-ended cover so that only the breadsticks’ ends protruded from these covers while their broken and intact middle sections were hidden from the subject’s view by being underneath the cover. The ends of the broken breadstick were arranged so that once the breadsticks were under the covers it looked identical to the intact breadstick with its naturally aligned ends. Subjects were then allowed to choose one breadstick and to consume it as a reward. We expected subjects to choose the intact breadstick over the broken breadstick because they knew which breadstick was under which cover and doing so allowed them to retrieve the full amount of breadstick whereas choosing the broken one would only allow subjects to retrieve less than half (hereafter half). Only subjects with a significant preference for the intact breadstick proceeded to the test conditions.

### Methods and procedure

#### Ethics declaration

All methods were performed in accordance with the relevant guidelines and regulations. The research was approved by an internal ethics committee at the Department of Comparative Cultural Psychology of the Max Planck Institute for Evolutionary Anthropology (MPI EVA), Leipzig, Germany, and is reported in accordance with ARRIVE guidelines. All experiments were non-invasive and strictly adhered to the legal requirements of Germany. Animal husbandry and research complied with the European Association of Zoos and Aquaria’s (EAZA) Minimum Standards for the Accommodation and Care of Animals in Zoos and Aquaria and the World Association of Zoos and Aquariums’ (WAZA) Ethical Guidelines for the Conduct of Research on Animals by Zoos and Aquariums. Informed consent was obtained from the legal guardians of all subjects for the publication of identifying information, including images and video samples, in an online open-access publication. The Wolfgang Köhler Primate Research Centre (WKPRC) of the MPI EVA at Leipzig Zoo, where all subjects were housed and the study was conducted, consented to their participation in the study and to the use of identifying information in this publication. The experimenter (M.N.S.) also agreed to the publication of her identifying information.

#### Subjects

Twelve subjects, seven Western chimpanzees (*Pan troglodytes verus*; 4 females, 3 males; Mean age = 28 years) and 5 Sumatran orangutans (*Pongo abelii*; 4 females, 1 male; Mean age = 30 years), participated in this first familiarisation phase (see Table 1). Eleven subjects were adults and one was a juvenile. Each subject was tested individually in a dedicated indoor testing room and was free to discontinue participation at any time. No subject was deprived of food or water during the study. Three females (Natascha, Padana, and Raja) had dependent infants who could not be temporarily separated from their mothers during testing and therefore these infants remained present during testing but were not included as subjects themselves. To ensure they were comfortable, the infants could freely move between the test and an empty adjacent compartment through a small gap in the door between the two. The youngest subject, Azibo, was only comfortable to participate in testing when another test subject was present in the adjacent compartment. The door between the two compartments was closed and we prevented the waiting chimpanzee from watching the test procedure by placing large black occluding panels on the wall between the compartments.

All subjects had previously participated in a novel information-seeking metacognition task with a similar test setup using breadsticks. This previous study had not involved subjects inferring the broken or intact properties of the breadsticks.

#### Materials

The test apparatus consisted of a grey sliding platform (79.5 cm x 33 cm) that rested on a test table that was mounted onto and flush with the front of the subject’s test compartment. An opaque light-blue Perspex tube was cut longitudinally through its center, which resulted in two half tubes (12.5 cm x 5 cm) that were used as covers to hide the properties of the breadsticks. Each cover was placed on the sliding platform, 31 cm apart and 1.5 cm from the sliding platform’s front that faced the subject. One intact breadstick (21.5 cm) was used as the optimal reward and a second broken breadstick was used as the sub-optimal reward. The broken breadstick was made by removing a 5.5 cm section so that two halves of equal length (8 cm) remained. During testing, the subject sat or stood behind a transparent Plexiglas window, which contained two circular openings (2 cm in diameter) through which the subjects could extend their fingers to indicate a choice.

#### Procedure (also see supplementary video 1)

The sliding platform was retracted (10 cm) from the test compartment’s window front, which was out of the subject’s reach. The experimenter (E) presented the covers and breadsticks to the subjects by placing them horizontally on the sliding platform, the covers on the left and right and the breadsticks in the middle (the broken one in front of the intact one), so that both the covers and breadsticks were oriented in parallel to both the front edge of the sliding platform and the Plexiglas window. The E then placed the intact breadstick in front of one cover and the broken one, with an obvious gap (5.5 cm) between its two sections, in front of the other cover so that subject could clearly see the properties of each breadstick. The E then placed the covers over the breadsticks’ middle sections so that their properties (intact vs. broken) were hidden. The breadsticks were positioned against the front walls of the covers (from the subject’s view), and both ends of each breadstick protruded from the covers so that each breadstick appeared as equally long. After 5 s, the E pushed the sliding platform towards the subject so that it was flush with the test compartment’s window front, and the outer ends of the breadsticks were positioned in front of the window’s two circular openings. This allowed subjects to choose one breadstick by extending their fingers through the respective opening to point to or touch the breadstick’s outer end (see supplementary video sample 1 for the full test procedure). A choice was scored as the first opening (left or right) of the window through which the subject extended a finger. After the subject’s choice, the E picked up the chosen breadstick and gave it to the subject. If subjects chose the intact breadstick, they would receive the whole breadstick whereas if they chose the broken one, they would only receive half. The test trials were presented in a pseudorandom manner with the rule that the intact breadstick did not appear on the same side on more than three consecutive trials. Each subject received a total of 12 familiarisation trials conducted in a single session on the same day. The criterion for passing the familiarisation phase and for proceeding to the experimental phase was that each subject chose the intact breadstick on at least 10 of the 12 trials.

### Data scoring and analysis

Binomial tests conducted in SPSS 28 were used to determine whether each subject passed the familiarisation phase. All familiarisation, test and control trials of this study were video recorded, and inter-rater reliabilities were independently scored in 50% of the trials of each subject from the video clips and statistically determined using Cohen’s Kappa^22^. Inter-rater reliabilities were excellent for the subjects’ performance scores in all experiments (familiarisation and experiments 1–9: *Kappa* = 1.00; *p* <.001; experiment 10: *Kappa* = 0.96).

### Results

All subjects passed the familiarisation phase by showing a clear preference for the intact breadstick: two subjects chose it in 10 trials (Azibo and Bimbo; Binomial tests: *p* <.05), one subject in 11 (Raja; Binomial test: *p* <.006) and the remaining 9 subjects chose the intact breadstick in all trials (Binomial tests: *p* <.001); see Table 1.

**Table 1:**
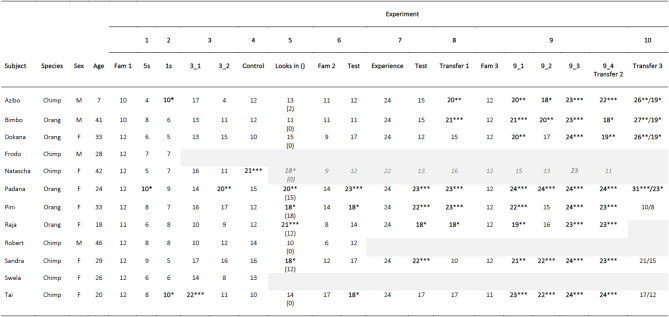
Number of trials in which subjects chose the intact breadstick when it was paired with a broken one in experiments 1–10. Trial numbers: 12 in familiarisations (fam) 1 and 3, and exp 1 and 2; 24 in experiments 4–9 and fam 2; and 32/24 in exp 10. Choices rewarded: fam 1 and exp 1–5, intact and broken breadstick; fam 2 and 3 and exp 6–10, only intact breadstick. Excluded subjects (dropouts) are indicated by grey cells, including Natascha who was excluded from the sample in exp 5. Significance levels: *P <.05, **P <.01, ***P <.001.

### Discussion

All subjects clearly preferred the intact breadstick over the broken one. Therefore, all subjects participated in the following experiment in which we assessed if they could infer the breadsticks’ properties by using indirect information to choose the intact breadstick over the broken one.

## Experiment 1: can orangutans and chimpanzees infer the breadsticks’ properties after observing indirect information about the properties of breadsticks?

We investigated if orangutans and chimpanzees can infer the properties of the breadsticks when they are given indirect visual information about the differences in the breadsticks’ hidden properties (intact vs. broken). The indirect visual information consisted of two visual cues: the temporary movement of each breadstick’s outer end. We predicted that if subjects could infer the properties of the two breadsticks, they would be more likely to choose the intact breadstick over the broken one.

### Methods and procedure

#### Subjects

The same twelve subjects who had participated in the previous familiarisation phase were tested.

#### Materials and test procedure

**Fig. 1:**
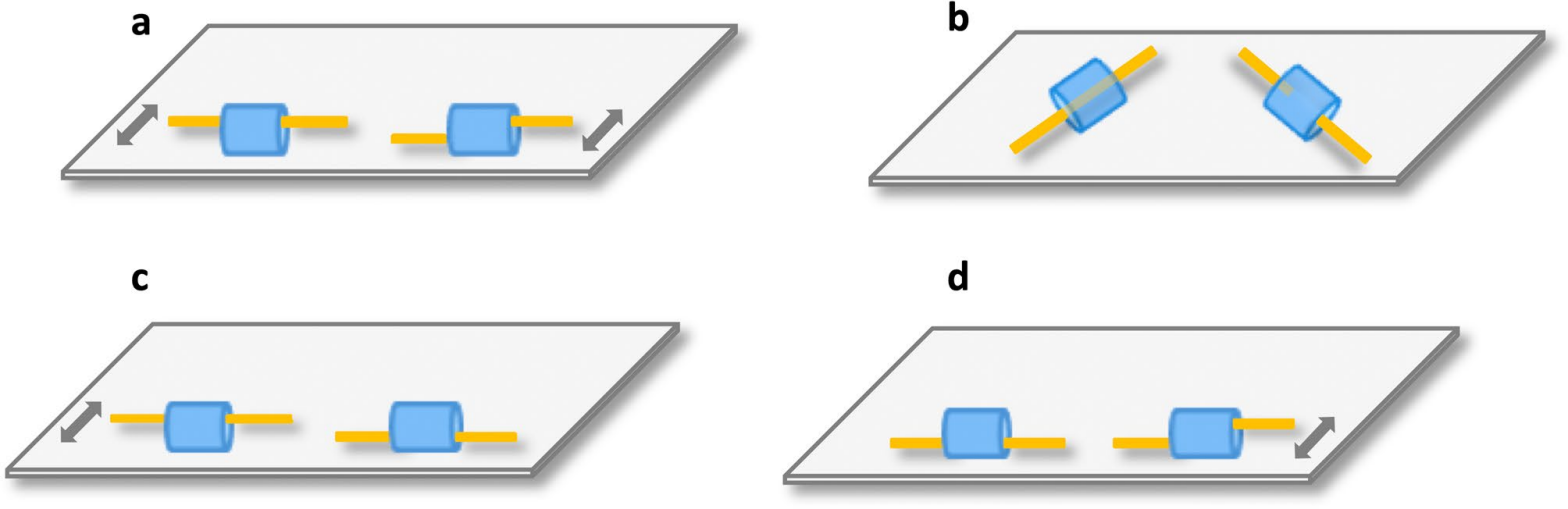
The temporary visual cues provided in experiments 1,2, 5, 6, and 7 (**a**); the additional full visual access in experiment 5 (**b**); and the single temporary visual cues in the two conditions of experiment 3 (**c**,** d**). Two ‘slide’ cues per trial (**a**): both breadsticks’ outer ends are sequentially moved (i.e., slid backwards and forwards into their original position); this also moves the intact but not the broken breadstick’s inner end. Looks possible (**b**): after the two indirect cues (**a**), both breadsticks and covers are turned diagonally allowing subjects to obtain full visual information on the breadsticks’ properties by peeking into the covers. **(c**, **d**): same ‘slide’ cue but only the intact breadstick is moved in condition 1 (**c**) and only the broken one is moved in condition 2 (**d**). *Note*: Shown is the view from the subject’s perspective. For demonstrative purposes, the intact breadstick is depicted on the left side when in fact its position was counterbalanced between the left and right side.

The set up and procedure were similar to the familiarisation condition. The only differences were that after presenting the empty covers and the two breadsticks to the subject, the E vertically placed an opaque panel (a thin acrylic black sheet: 90 × 50 cm) between the sliding platform and the test compartment’s window. This panel prevented subjects from seeing which breadstick was placed under which cover. When the occluding panel was removed subjects therefore did not know which one was the intact or broken breadstick. Before allowing subjects to choose one breadstick, the E indirectly demonstrated the breadsticks’ properties by sliding each breadstick’s outer end from the cover’s front to its back wall before sliding the breadstick end back in its original aligned position. The E always moved the breadstick that was to her left first, and the intact or broken breadstick did not appear on the same side in more than three consecutive trials. This procedure meant that subjects could not be successful by simply using a rule of choosing the first or last breadstick that the E touched. Moving the outer breadstick ends would cause the broken breadstick’s inner end to remain stationary and result in temporal dealignment (about 2 s) of the two breadstick ends, whereas moving the intact breadstick’s outer end in the same way would cause both breadstick ends to move simultaneously and thus remain aligned (Fig. 1a and supplementary video 2). Therefore, subjects could infer which breadstick was broken and intact. Once the second breadstick had been moved, the E waited 5 s before pushing the sliding platform towards the testing window to allow the subject to choose one of the breadsticks. The subjects received one test session of 12 trials.

### Data scoring and analysis

In this, and in all following experiments and controls, individual-level binomial tests conducted in SPSS 28 were used to determine whether subjects chose the intact breadstick significantly more often than one would expect by chance. In addition, we fitted a binomial generalised linear mixed model (GLMM) with a logit function to estimate whether the probability of choosing the intact stick (yes, no) co-varied with species, trial number, and age. Subject was included as random factor in all models. To test the significance of predictors as a whole, we compared the fit of the full model with that of the null model comprising only random factors^23,24^. All models were conducted in R (see model results in supplementary tables, data file 1 and code script).

### Results

One of the 12 subjects chose the intact breadstick in 83% of the test trials, which was significantly more often than expected by chance (Padana; Binomial test: *p* =.039), whereas none of the other subjects choose the intact breadstick more often than by chance (Table 1). The GLMM confirmed that species (*p* =.410), trial number (*p* =.445) and age (*p* =.379) did not significantly affect the subjects’ test performance (likelihood ratio test full-null model comparison: *X*^*2*^ = 1.26, *df* = 2, *p* =.531; Supplementary Table S1).

### Discussion

Only one of the 12 subjects consistently chose the intact breadstick suggesting that this subject could infer its properties. However, this finding is puzzling considering that all other subjects failed to show success in the task. One possibility for the subjects’ poor performance was that the 5 seconds waiting time after they had observed the breadstick being moved, and before they could make a choice, was too long. To infer the breadsticks’ properties, subjects would have to encode the differences in each breadstick’s movement and then remember the inferred location of the broken or intact breadstick. Having a 5 s delay may have resulted in subjects forgetting the location of the broken or intact breadstick, which could have been compounded by having the breadsticks’ ends protruding out of the covers. Subjects may have been distracted during the delay by looking at the desirable ends of the food, which contributed to them forgetting the inferred location of each breadstick type. We addressed this possibility in the next experiment by repeating experiment 1, but reducing the task demands by removing the 5 s delay.

## Experiment 2: can orangutans and chimpanzees infer the breadsticks’ properties after observing relevant indirect information when they can immediately make their choice?

We explored if orangutans and chimpanzees can infer the breadsticks’ properties when they can immediately choose a breadstick after having been shown indirect visual information about the differences in the breadsticks’ hidden properties. Our prediction was that if the time delay had masked the subjects’ presumed inferential abilities, those subjects who had been unsuccessful in experiment 1 should now be successful. We also predicted that the one subject who had been successful in experiment 1, should be equally or even more successful in the absence of the short time delay.

### Subjects

We tested the same subjects who had participated in experiment 1.

### Materials and test procedure

The set-up and procedure were almost identical to experiment 1 (Fig. 1a). The only difference was that after indirectly demonstrating the breadsticks’ hidden properties to the subject, the E immediately pushed the sliding platform towards the subject so a choice could be made, i.e., there was no 5 s delay.

### Data scoring and analysis

Data scoring and statistical analyses were the same as in experiment 1.

### Results

Two of the twelve subjects chose the intact breadstick significantly more often than by chance by doing so in 83% of the test trials (Tai and Azibo; Binomial test: *p* =.039), whereas none of the other subjects were successful (Table 1). A GLMM confirmed that species (*p* =.633), trial number (*p* =.688) and age (*p* =.074) did not significantly affect performance (likelihood ratio test full-null model comparison: *X*^*2*^ = 0.39, *df* = 2, *p* =.823; Supplementary Table S2).

### Discussion

Contrarily to our prediction, only two individuals (both chimpanzees) were successful in the task when no time delay was imposed. Equally unexpectedly, the only individual (an orangutan) who had been successful in experiment 1 was no longer successful in what we had predicted to be a less demanding task version. These findings indicated that the task was still too difficult for most individuals and that task-related factors other than the short delay may have made the task too demanding. One possibility was that having to attend to the sequential movement of both breadsticks might have distracted the subjects or prevented them from simultaneously keeping the full experimental setup in mind. In the next experiment, we therefore further reduced the attentional and working memory demands by reducing the amount of breadstick movement.

## Experiment 3: can orangutans and chimpanzees infer the properties of breadsticks after seeing indirect information about the properties of only one of two breadsticks?

We investigated if reducing the demands of the task used in experiment 1 and 2 would improve the subjects’ performance. This was achieved by reducing the indirect visual cues from two to one by moving either the intact or the broken breadstick rather than both breadsticks in each test trial.

### Methods and procedure

#### Subjects

We tested the same 12 subjects who had participated in experiment 2.

#### Materials and test procedure

The test-set up was identical to experiment 2 and the procedure only differed in that instead of moving both breadsticks and providing two indirect visual cues regarding the breadsticks’ properties, the E now only provided a single cue: only moving the outer end of one breadstick. There were two test conditions that differed in which breadstick was moved by the E. In condition 1 (intact slid), only the intact breadstick was moved (Fig. 1c and supplementary video 3) whereas in condition 2 (broken slid) only the broken breadstick was moved (Fig. 1d and supplementary video 4). The two conditions were intermixed within test sessions so that subjects received 24 trials of each condition in 4 test sessions of 12 trials (6 trials per condition) each. Trials within sessions were pseudo-randomised with the rule that each test condition did not appear in more than three consecutive trials and the intact or broken breadstick did not appear on the same side in more than three consecutive trials.

### Data scoring and analysis

Data scoring and statistical analyses were the same as in experiment 1. However, in the binomial GLMM we included an additional fixed factor condition (i.e., which breadstick was moved). We fitted whether an individual chose the intact breadstick (yes, no) as response and species, condition (intact slid or broken slid), trial number, and age as fixed factors, and subject was included as random factor.

### Results

One chimpanzee (Frodo) was excluded during testing because he consistently sat in the far corner of the test window rather than in its middle and did not fully attend to the breadstick and cover that were presented farthest away from him.

Two of the 11 subjects who completed testing successfully chose the intact breadstick more often than expected by chance in one of the two test conditions, whereas no other subject passed any test condition (Table 1). One of the two successful subjects chose the intact breadstick significantly above chance levels in the intact slid condition by doing so in 92% of the test trials (Tai; Binomial test: *p* <.001) but not in the broken slid condition, in which she chose the intact breadstick in 46% of the trials (*p* =.839). The other successful subject chose the intact breadstick significantly above chance levels in the broken slid condition by doing so in 83% of the test trials (Padana; Binomial test: *p* =.002), but not in the intact slid condition, in which she chose the broken breadstick in 58% (*p* =.541) of the test trials. A GLMM with overall performance as response variable showed a significant effect of test condition in that subjects performed better in the intact slid than the broken slid condition (*p* =.013), whereas the other fixed effects did not affect the subjects’ performance (species: *p* =.704; trial number: *p* = 214; age: *p* =.816, likelihood ratio test full-null model comparison: *X*^*2*^ = 7.93, *df* = 3, *p* =.048; Supplementary Table S3).

### Discussion

We found that only two subjects (one chimpanzee and one orangutan) were successful when in each test trial only one of the two breadsticks was moved. Interestingly, the only subject (a chimpanzee) who was successful when only the intact breadstick was moved, was unsuccessful when only the broken breadstick was moved. And the opposite was found for the subject (orangutan) who was successful when only the broken breadstick was moved but not when only the intact breadstick was moved. Both successful subjects had been successful in one of the previous two experiments. If subjects were inferring the properties of breadsticks, it would be expected that they would show success in both the broken slid and the intact slid conditions, but both subjects failed one of the two conditions. Moreover, one subject was only successful in the broken slid condition, which is presumably more difficult than the intact slid condition because subjects are required to infer by exclusion the location of the intact breadstick. It is possible that the subjects who were successful were not inferring the properties of the breadsticks but could detect and remember the extremely slight differences between the two breadsticks because sometimes there were slight variations in the shades of colour along the breadsticks’ lengths, a natural result of the baking process.

It could be argued that if subjects could use such slight differences, then they would have been successful in both the intact slid and the broken slid conditions. However, keeping track of slight visual differences between the breadsticks would likely require a great deal of attention and concentration that would be probably difficult to maintain across all test trials and therefore would result in various degrees of individual success, such as we found in this experiment.

In the next experiment we tested if subjects could detect slight differences between the two breadsticks. However, rather than only testing the two subjects who had shown success, we tested all subjects who had participated in experiment 3 because it was possible that they could detect the slight differences between the breadsticks but were more prone to distraction.

## Experiment 4: can orangutans and chimpanzees visually distinguish the breadsticks in the absence of indirect visual information about the properties of breadsticks?

This experiment served as a control to ascertain that subjects were unable to simply base their choices on slight visual differences between the breadsticks. We again presented the subjects with the partially hidden breadsticks in the control trials but contrarily to the test conditions, the E did not move any breadstick so that the subjects received no indirect visual information about the breadsticks’ properties on which they could base their choices. Subjects could now only be successful if they were able to notice very slight differences in the breadsticks’ physical appearance when they were first presented without covers and track them during testing. We expected subjects to choose the breadsticks at random in the control trials unless they could detect such slight visual differences.

### Subjects

We tested the same 11 subjects who had completed experiment 3.

### Procedure (also see supplementary video 5)

The set up and procedure corresponded to the ones in experiment 2 with the only difference being that after partially hiding the breadsticks and removing the occluding panel, the E did not move any breadstick before pushing the sliding platform towards the subjects so they could make their choice. Subjects received two daily control sessions of 12 trials each, which resulted in a total of 24 control trials.

### Data scoring and analysis

Data scoring and statistical analyses were the same as in experiment 1.

### Results

Ten of the 11 subjects performed at chance-levels in the control trials by choosing the intact and broken breadstick equally often (Binomial tests: *p* >.05), whereas one subject (Natascha) significantly chose the intact breadstick more often than expected by chance (i.e., in 88% of the trials; Binomial test: *p* <.001; Table 1). A GLMM showed that trial number had a significant effect on the subjects’ success in that their performance decreased across control trials (*p* =.038), whereas species (*p* =.133) and age (*p* =.103) did not affect performance (likelihood ratio test full-null model comparison: *X*^*2*^ = 6.47, *df* = 2, *p* =.039; Supplementary Table S4).

### Discussion

Our findings revealed that 10 of the 11 subjects were unable to visually distinguish the partly hidden breadsticks in the absence of any experimenter-given visual cues (i.e., when none of the two breadsticks were moved). However, one subject (Natascha) was successful suggesting that she was able to visually distinguish the breadsticks solely based on subtle differences in their physical appearance. Curiously, Natascha had not passed the inference tasks in the previous three experiments. But if she had the ability to distinguish subtle differences between the breadsticks, then as discussed earlier, it may have been much more difficult for her to keep track and remember the location of each breadstick’s intact or broken state when the E moved them. For the remaining experiments we decided to continue testing Natascha but her performance was not included in the analyses of the subjects’ performance in each experiment. This allowed us to explore how her performance would develop if she was using a tracking strategy (i.e., if she could consistently apply her supposedly direct visual strategy). We found that Natascha only passed one more experiment (5) and one condition of experiment 9 suggesting that if she was tracking the breadsticks, she found it difficult to do this consistently.

None of the three subjects who had been successful in the inference tasks in the previous experiments could detect subtle differences between the breadsticks therefore suggesting that they had inferred the breadsticks’ properties in the previous experiments. Yet, 7 subjects showed no evidence of this inference ability. However, it is possible that these subjects had this ability, but they failed to express it because they were limited by other factors. One possibility was that the unsuccessful subjects were facing a problem of recall because subjects were required to infer the breadsticks’ properties from the indirect cues that were given and then remember which hidden breadstick was intact or broken. One way to investigate if it was a problem of recall, was to give subjects the opportunity to seek additional information that would inform them about the properties of each breadstick. We addressed this in the next experiment by allowing subjects the opportunity to seek direct visual information about the breadsticks’ properties by allowing them to look inside the front openings of the covers once they had seen the indirect visual information.

## Experiment 5: can orangutans and chimpanzees infer the breadsticks’ properties based solely on indirect visual information or do they seek direct visual information when possible?

We investigated if subjects could infer which breadstick was intact after seeing indirect information about both breadsticks’ hidden properties. However, after presenting the subjects with the same indirect visual information as in experiments 1 and 2 (moving the intact as well as the broken breadstick), we gave subjects the opportunity to seek direct visual information about the breadsticks’ properties by looking inside the covers. If subjects had failed the previous inference tasks because they had problems of recalling which breadstick was intact or broken when indirect cues were given, we expected that they would look inside the covers more often than subjects who had not failed because these subjects might be inferring this information without incurring the added cost of bending down and looking inside one or both covers.

### Subjects

We tested 10 subjects who had completed experiment 4 (Natascha was excluded from the sample). All subjects had experience of searching for the breadsticks’ properties by looking inside covers at breadsticks in a metacognitive study that had been conducted prior to the current study.

### Test procedure

The procedure and setup largely corresponded to the one used in experiment 1, but we made the following changes. The breadsticks and covers were placed on two small grey plastic sheets (23.5 cm x 13 cm) at a distance of 1 cm from the sheets’ front edges. The E placed the breadsticks in the covers’ centers and after removing the occluding panel, the E slid each breadstick’s outer end first backwards towards the cover’s back wall, then forwards towards its front wall and finally back into its original centered position. The E then simultaneously slid both sheets, with the partly covered breadsticks on top, diagonally to an angle of approximately 60 degrees (see Fig. 1b). Positioning the partly covered breadsticks in this way would allow the subjects to look into the front openings of the covers by bending down and peeking into the covers to see whether the respective breadstick was intact or broken (supplementary video 6). After 5 s had elapsed, the E pushed the sliding platform towards the subject who could make a choice. Subjects received a total of 24 trials in two daily sessions of 12 trials each.

### Data scoring and analysis

The subjects’ individual looking behaviour in each test trial was coded from the video clips. A ‘look’ was scored as the subject bending down and peeking into the cover. The inter-rater reliability for the looks was excellent (Kappa = 0.93; *p* <.001). Statistical analyses of subjects’ performance were the same as in experiment 1. Additionally, we included the number of looks in the binomial GLMM, in which we estimated whether a subject chose the intact stick (yes, no) as response and species, number of looks, trial number, and age as fixed factors.

### Results

One subject (chimpanzee Swela) had to be excluded as she was no longer motivated to participate. Four of the remaining 9 subjects chose the intact breadstick significantly above chance levels, with the proportion of intact-breadstick choices ranging from 88% (Raja; Binomial test: *p* <.001) and 83% (Padana; Binomial test: *p* =.002) to 75% (Pini and Sandra; Binomial tests: *p* =.023). All four successful subjects looked inside the covers on between 12 and 18 occasions (Table 1). One subject (Azibo), who was unsuccessful, also looked inside the covers but he only did so on two occasions.

A GLMM revealed the number of looks (*p* =.001) and trial number (*p* =.004) predicted the probability of choosing the intact breadstick (likelihood ratio test full-null model comparison: *X*^*2*^ = 17.10, *df* = 3, *p* =.001; Supplementary Table S5). Although the subjects’ overall performance decreased across trials, those subjects who looked inside the covers were more likely to choose the intact breadstick than those who did not. Species (*p* =.125) and age (*p* =.063) did not influence performance.

### Discussion

We found that four subjects, three orangutans and one chimpanzee, successfully chose the intact breadstick. One of the successful orangutans had previously passed experiment 1 and condition 2 (broken slid) of experiment 3, whereas the other two orangutans and the chimpanzee had not passed any previous experimental conditions that involved inferring the breadsticks’ properties. All successful subjects searched for additional information about which breadstick was intact by bending down and looking into the covers. This was an expected finding, in line with a recall problem of the indirect visual information, for the previously unsuccessful subjects, yet an unexpected finding for the subject who had shown evidence of inferring the breadsticks’ properties in previous experiments. If this subject could infer which breadstick was intact from indirect cues, it is surprising that she incurred the added cost of bending down to look under the covers when it was not necessary. One possibility is that she was just rechecking that her inferences were correct (e.g., the passport effect^25^).

The subjects who failed the current experiment provided little evidence that they had any problem recalling the information provided by the E’s indirect cues to which breadstick was intact. If they did have a problem recalling such information, it would have been expected that these subjects would have searched inside the covers for additional information to establish which breadstick was intact. However, none of these subjects searched the covers in any of the trials. However, it is curious why these subjects forfeited the chance to search inside the covers as this would have guaranteed them the chance to retrieve the intact breadstick on every trial. One possibility is that the unsuccessful subjects in this, and previous experiments, were not motivated to engage in solving the inference tasks because they always received a reward no matter what they chose because choosing the broken breadstick would still allow them to consume half of a reward. It is therefore possible that this reward contingency scheme interfered with the subjects’ choices and this would explain why the previous experiments had shown only a few subjects correctly choosing the intact breadstick. We addressed this possibility in the next experiment by not rewarding subjects if they chose the broken breadstick.

## Experiment 6: does the subjects’ performance of inferring the breadsticks’ properties improve when only one of two possible breadstick choices is rewarded?

We investigated whether the subjects’ ability to select the intact breadstick would improve, considering that choosing the broken breadstick would incur an additional cost, as subjects would not receive any reward if they made this choice. Prior to the test condition, we familiarised subjects with the new reward-contingency scheme, where they only received the breadstick if they selected the intact one and received nothing if they chose the broken one.

### Familiarisation: subjects experience that they only receive a reward if they choose the hidden intact breadstick

Subjects were given experience that the E would only give them the intact breadstick if they chose it, whereas they would not receive anything if they chose the broken one. As in experiment 4, the E did not move any of the breadsticks meaning that subjects were given no indirect information about which breadstick was intact or broken. It was therefore expected that subjects would choose at random meaning that all subjects should experience that they would not receive any breadstick reward unless they chose the intact one.

#### Subjects

The same nine subjects who had completed experiment 5 participated in the familiarisation trials and the test condition.

#### Procedure

The set up and procedure corresponded to the ones used in experiment 4 in which the breadsticks were hidden inside the covers and no indirect information was given that subjects could use to infer the properties of each breadstick. The only change to the procedure was that if the subject chose the broken breadstick, the E quickly retracted the sliding platform and removed both breadsticks without giving any breadstick to the subject. Each subject received a total of 24 familiarisation trials conducted in 2 daily sessions of 12 trials each.

### Data scoring and analyses

As in the first familiarisation, we used Binomial tests to assess whether each subject’s performance differed from chance level.

### Results

None of the subjects significantly chose the intact breadstick above chance levels. Subjects chose the broken breadstick in a minimum of 29% (Tai) and a maximum of 75% (Robert) of the trials (Table 1).

### Discussion

All subjects experienced to various degrees that they would not receive any breadstick reward unless they chose the intact breadstick. All subjects participated in the following test condition.

### Test condition

We investigated if increasing the cost of choosing the broken breadstick would improve the subjects’ performances at inferring which breadstick was broken or intact. As in the familiarisation trials, the E only gave subjects the intact breadstick if they chose it, whereas she gave them nothing if they chose the broken one.

#### Procedure

The test set up and procedure were similar to the ones in experiment 2 in which the E had moved both breadsticks (Fig. 1a). The only difference was that subjects only received a reward if they chose the intact breadstick. If subjects chose the broken breadstick, the E immediately retracted the sliding platform out of the subject’s reach and removed both the broken and intact breadstick (supplementary video 7). Each subject received a total of 24 trials conducted in two daily sessions of 12 trials each.

### Data scoring and analyses

Data scoring and statistical analyses were the same as in experiment 1.

### Results

Three of the nine subjects successfully chose the intact breadstick in 96% (Padana; Binomial tests: *p* <.0001) and 75% of the test trials (Pini and Tai; Binomial test: *p* =.023), respectively (Table 1). Species (*p* =.350), trial number (*p* =.133), and age (*p* =.504) had no significant effect on performance (GLMM; likelihood ratio test full-null model comparison: *X*^*2*^ = 3.12, *df* = 2, *p* =.209; Supplementary Table S6).

### Discussion

Increasing the cost of choosing the broken breadstick had little effect on the subjects’ performance of inferring the breadsticks’ properties. Only three of the 9 individuals, two orangutans and one chimpanzee, showed evidence of inferring which breadstick was intact or broken, and two of these were the same individuals who had previously passed some of the test conditions in experiments 1 (Padana), 2 (Tai), and 3 (Padana and Tai) in which they had also received indirect visual information about the breadstick properties. Our results do not support the idea that subjects failed to infer the breadsticks’ properties in previous experiments simply because they always received half of the breadstick when choosing the broken one. Had this been the case, we would have expected subjects to now infer the breadsticks’ properties because doing so enabled them to receive the intact breadstick whereas broken breadstick choices remained unrewarded. This only remained a possibility for one of the three subjects (Pini) who had been successful for the first time in the previous experiment (experiment 5) when obtaining full visual information and who was now still successful when this was no longer possible.

The results of our study so far revealed that only a minority of subjects showed evidence that they can infer the breadsticks’ properties. However, it is possible that the subjects in this study do not have the ability to make such inferences and those who showed success may have been using heuristics. Alternatively, it is possible that successful subjects do have the ability to infer the breadsticks’ properties, but this success depends on relevant experience. It should be noted that only one of 12 subjects had passed the first inference task of this study and the experience of that experiment and subsequent experiments may have helped some subjects to infer the breadsticks’ properties. For subjects who were consistently unsuccessful, it is possible that they require more explicit experience to successfully engage in such tasks. We addressed this possibility in the next experiments. (Note that, although we found little evidence that the new reward scheme influenced the subjects’ motivation or performance, we continued rewarding only intact-breadstick choices for the remainder of the study. This ensured consistency with the new reward scheme and ruled out the possibility of subjects developing motivational issues.)

## Experiment 7 does the subjects’ performance improve after experiencing the mechanism of each breadstick when they are moved?

We explored if the subjects’ failure rates in our original breadstick inference task (e.g., experiment 1), in which both breadsticks had been moved and the subjects had only received indirect visual information about the breadsticks’ properties, would improve after receiving complete visual information about the mechanism of the breadsticks’ movement. We expected that once subjects had observed and understood this mechanism, they would be able to apply their new knowledge to the original inference task and their performance would improve. We decided to include the subjects who had been successful in the original inference task (experiment 2), so that all subjects received the same experience. We did this in case the subjects’ success was not a result of being able to infer the breadsticks’ properties, but it was a result, for example, of using heuristics, which follow-up experiments in this study would address. Prior to the test condition, in which they would be presented with the original inference task (experiment 2), all subjects received direct visual information of the mechanism in place.

### Experience phase: subjects were shown the mechanism of the breadsticks’ movement

We provided subjects with complete information of how moving the breadsticks’ ends affected their intact or broken properties differently, i.e., that when the intact breadstick’s outer end was moved, this would cause the full breadstick, including its inner end, to move whereas when the broken breadstick’s outer end was moved, this would cause the breadstick’s inner end to remain stationary. After showing subjects this information, the breadsticks were covered to hide their properties and subjects were allowed to make a choice. We expected all subjects to succeed in choosing the intact breadstick when it was hidden again as they only had to remember the location of the broken and intact breadstick. Only successful subjects would then proceed to the test condition.

#### Subjects

The same nine subjects as in experiment 6 participated in the experience phase and the test condition. Each subject participated in two sessions of 12 trials per day (*N* = 24) in both the experience phase and the test condition.

#### Procedure

The set up and procedure in the experience phase largely corresponded to experiment 6 with the exceptions that before moving each breadstick, the E lifted the cover and held it up while moving the breadstick’s outer end so that the subject could clearly see how this would cause the intact breadstick to move in its entirety whereas moving the outer end of the broken breadstick would not move its unattached end. After moving each breadstick, the E replaced the cover so that the middle sections of both breadsticks were concealed when the subject made their choice (supplementary video 8). Subjects received 24 experience trials.

### Data scoring and analyses

As in the familiarisation phases, we used Binomial tests to assess whether each subject’s performance differed from chance level.

### Results

Robert only participated in session 1 of the experience phase because he sadly passed away and was therefore excluded from the analyses. All 8 subjects who completed the experience phase chose the intact breadstick in 100% of the experience trials, in which they could directly observe the mechanism of the breadstick movement (Table 1).

### Discussion

All subjects passed the experience phase and went on to participate in the test condition, in which the breadsticks were once again hidden and subjects were only provided with indirect visual information about each breadstick’s properties.

### Test condition: subjects were tested with the original task

We investigated if the subjects could apply the new knowledge of the mechanism of the breadstick movement they had gained in the experience phase to the original task, in which the breadsticks’ properties were hidden. We expected that having observed the mechanisms involved when the breadsticks were moved would help the subjects to be more successful in the test condition, in which they had to infer which breadstick was intact and which one was broken based on indirect rather than direct visual information.

#### Procedure

The set up and procedure were identical to the ones used in experiment 6. Both breadsticks had their middle sections covered again and the E moved the outer breadstick ends before re-aligning them with the inner ends and only rewarded the subjects if they chose the intact breadstick.

### Data scoring and analysis

Data scoring and statistical analyses were the same as in experiment 1.

### Results

Four of the 8 subjects chose the intact breadstick significantly above chance levels in the test condition: one subject in 96% (Padana; Binomial test: *p* <.001), two subjects in 92% (Pini and Sandra; Binomial test: *p* <.001) and one subject in 75% (Raja; Binomial test: *p* =.023) of the test trials (Table 1). None of the fixed effects significantly affected performance (likelihood ratio test full-null model comparison: *X*^*2*^ = 0.02, *df* = 2, *p* =.988; species: *p* =.992; trial number: *p* =.878; age: *p* =.911; Supplementary Table S7).

### Discussion

The four subjects (three orangutans and one chimpanzee) who passed the test condition were again those who had also passed some of the previous experiments. However, one successful orangutan (Raja) and one successful chimpanzee (Sandra) had previously only passed experiment 5, in which they had looked inside the covers in some test trials and therefore not shown evidence of inferring the breadsticks’ properties in any of the previous experiments. This suggests that observing the mechanism of the breadsticks’ movement in the experience phase enabled these two subjects to understand the properties of the hidden breadsticks when they were indirectly given cues by the E. However, it is unclear if their success was based on being able to infer the breadsticks’ properties or if they simply learnt to select the intact breadstick by simply remembering from the experience trials how each breadstick acted when it was moved. In the next experiment we therefore investigated if subjects could infer the breadsticks’ properties by introducing a novel way of indirectly showing the subjects which breadstick was broken or intact.

## Experiment 8: can orangutans and chimpanzees infer the breadsticks’ properties in a different novel task?

We introduced a novel breadstick task to explore whether those subjects who had shown evidence of inferring the breadsticks’ properties, in at least some of the previous experiments, could apply their knowledge to a new situation. Subjects were presented with a new type of indirect information: instead of sliding the breadsticks backwards and forwards, they were now pushed into the covers before pulling them back into their original position. This new test set-up also allowed us to investigate whether previously unsuccessful subjects would be better able to use these new cues to infer which breadstick was intact. We were also interested in whether subjects who had previously succeeded could apply their experience to novel cues. For these subjects, this experiment served as a transfer test.

### Subjects

We tested the same 8 subjects who had completed experiment 7. Each subject participated in two daily sessions of 12 test trials each (*N* = 24).

### Test procedure

**Fig. 2:**
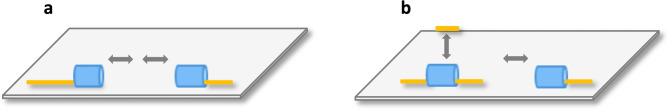
The two temporary visual cues in experiments 8 and 10. In experiment 8 (**a**), the inner ends of both the broken and intact breadstick are pushed inside and pulled back out of the covers; this only causes the intact but not the broken breadstick’s outer end to protrude from the cover. In experiment 10 (**b**), the middle piece is hidden under the same cover as the intact breadstick; the cover is then tilted and the middle piece temporarily lifted. The broken breadstick’s inner end is pushed inside the cover.

The set up and procedure were similar to experiment 6 with two important changes: the E moved the inner rather than the outer breadstick-ends and pushed the inner breadstick-ends inside the covers and pulled them back out into their original position. When she moved the intact breadstick in this way, its inner end temporarily disappeared under the cover whilst simultaneously causing its outer end to extend out from the outer opening of the cover before it was pulled back into its original position. Moving the broken breadstick’s inner end in the same way caused its inner end to temporarily disappear under the cover and its outer end to remain stationary before the breadstick was pulled back into its original position (Fig. 2a and supplementary video 9).

### Data scoring and analysis

Data scoring and statistical analyses were the same as in experiment 1.

### Results

Five of the 8 subjects chose the intact breadstick more often than expected by chance: two subjects in 96% (Padana and Pini; Binomial test: *p* <.001), one in 88% (Bimbo; Binomial test: *p* <.001), one in 83% (Azibo; Binomial test: *p* =.002) and one in 75% of the test trials (Raja; Binomial test: *p* =.023); Table 1. There was a marginally significant effect of species in that orangutans performed slightly better than chimpanzees (*p* =.046), but the full-null model comparison was not significant (likelihood ratio test full-null model comparison: *X*^*2*^ = 4.69, *df* = 2, *p* =.096). Therefore, this result should be treated with caution. Trial number (*p* =.233) and age (*p* =.322) had no effect on performance (Supplementary Table S8).

### Discussion

We found that four orangutans and one chimpanzee were successful at reliably choosing the intact breadstick. The three female orangutans who successfully choose the intact breadstick in the tests condition were the same individuals who had previously passed experiment 7. However, the two males, an orangutan (Bimbo) and a chimpanzee (Azibo), who also chose the intact breadstick successfully in the test condition, had been unsuccessful in most or all previous experiments in which they were required to infer the breadsticks’ properties (Azibo had only passed experiment 2 and Bimbo none of the previous experiments). This suggests they were now able to successfully use the new indirect visual cues to pass the task. A possible explanation of why two additional subjects increased their performance in the current experiment is that the new type of indirect visual information was more salient to them, possibly because of the larger temporary visible asymmetry in the amount of breadstick that protruded from the covers in comparison to the original cues. In fact, moving the intact breadstick involved its end moving out of the cover whereas moving the broken breadstick did not. It could therefore be argued that when the end of the intact breadstick moved out of the cover it attracted the successful subjects’ attention more than the broken breadstick and this is why they were more likely to choose it. It is also possible that the same temporary asymmetry in breadstick movement had the opposite effect on the two unsuccessful chimpanzees and distracted them from applying their previous experience and success to this new test set-up. We addressed this issue in the next experiment. We also conducted transfer tests, in which the asymmetry in movement of the breadsticks’ ends was absent and subjects could not directly apply what they might have learned over the course of the previous experiments but had to infer the breadsticks’ properties based on a new scenario.

## Experiment 9: can orangutans and chimpanzees use indirect information to infer the breadsticks’ properties when (1) only the broken breadstick is pushed, and (2) when the indirect visual cues do not involve any asymmetry in breadstick movement?

We tested subjects with a similar task to the previous experiment with the only difference being that only the broken breadstick was moved. We predicted that if subjects had been successful in the previous experiment because they were attracted to the end of the intact breadstick moving out of the cover, then subjects would not be successful when this pronounced asymmetry was absent i.e., when only the broken breadstick’s inner end was moved. We also tested subjects with a control condition in which the E slid the broken breadstick backwards and forwards (condition 2 from experiment 3). We predicted that if subjects could infer the breadsticks’ properties, they should be successful in all conditions whereas if they were using heuristics learnt from our previous experiments, they would only show success in the control condition as they had been tested repeatedly with this condition as the broken breadstick was always slid backwards and forwards in the majority of all our previous experiments.

We also tested subjects with novel tests that did not involve the E moving the protruding ends of the breadsticks. Subjects could therefore not use any heuristics that they might have learnt during the previous experiments that had always involved moving one or both ends of the breadsticks. One novel condition involved the E temporarily lifting the broken breadstick’s cover so that subjects could see that the breadstick underneath was broken (the cover over the intact breadstick was never lifted). The second condition involved using a broken breadstick that included its middle piece, i.e., the broken breadstick consisted of 3 pieces instead of two with a gap in the middle. Once the breadsticks were hidden, with their ends protruding from the covers, the E would remove the broken middle piece (that was completely hidden under the cover) and show it to the subject and then replace it (no direct information was given about the intact breadstick). Although, subjects did not know the location of the broken or intact breadsticks, they could infer that the broken piece corresponded to the broken breadstick.

Before conducting the tests, we familiarised subjects with the new 3-piece breadstick. We expected that when subjects were shown the three pieces of the broken breadstick being aligned, so it closely matched the intact breadstick, they would choose the intact breadstick over the broken one because this would result in them receiving the whole breadstick whereas choosing the broken one would result in no reward.

### Familiarisation

#### Subjects

The same 8 subjects as in the previous experiment participated in the familiarisation phase and the subsequent test conditions: five orangutans and three chimpanzees.

#### Procedure

The set up and procedure were similar to the first familiarisation phase we had conducted at the beginning of the study. The critical additions were that the broken breadstick consisted of three pieces, the two ends and its middle piece. The E aligned the three pieces of the broken breadstick so that it looked almost identical to the intact breadstick and then presented the breadsticks to the subjects. There was no time delay between the E’s demonstration and the subject’s choice. Each subject received 12 familiarisation trials conducted in a single session on the same day.

### Data scoring and analysis

Data scoring and analyses were the same as in the previous familiarisation phases.

### Results

One subject, Tai, chose the intact breadstick in 11 familiarisation trials (score: 92%) whereas all other subjects chose it in all 12 trials (score: 100%); see Table 1.

### Discussion

All subjects passed the familiarisation, indicating their ability to understand that a breadstick consisting of three broken pieces that are aligned to look unbroken is still broken compared to the intact breadstick.

All subjects participated in the following test phase.

### Test conditions

We tested two conditions that had been used in previous experiments that involved the E moving one end of the protruding breadstick (1) 2-piece slid cue, and (2) 2-piece pushed cue. And we tested two conditions that were novel to the subjects that did not involve the E moving the ends of the protruding breadsticks: (3) 2-piece uncovered cue, and (4) 3-piece middle-piece shown cue.

#### Procedure

**Fig. 3:**
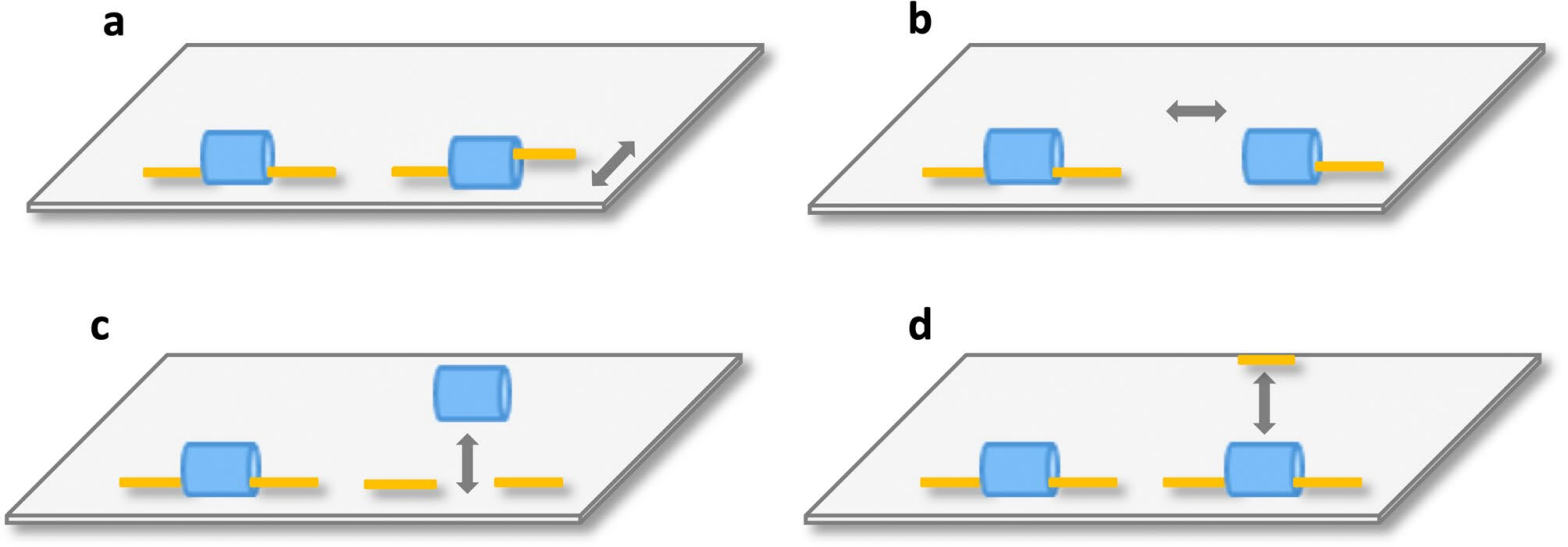
The temporary visual cues provided in experiment 9. The broken breadstick’s non-functional state is indicated by a 2-piece slid cue in condition 1 (**a**); a 2-piece pushed cue in condition 2 (**b**); fully revealed by a 2-piece uncovered cue in condition 3  (**c**); and indicated by the novel 3-piece middle shown cue in condition 4, in which a novel broken breadstick was introduced (**d**).

The setup of the 2-piece breadsticks conditions (1–3) corresponded to the one used in all the previous experiments (Fig. 3a-c). The procedure was also largely similar and only choices of the intact breadstick were rewarded. Condition 1: 2-piece slid cue; the E slid the broken breadstick’s outer end backwards and forwards into its original position (as in condition 2 of experiment 3; see Fig. 3a). Condition 2: 2-piece pushed cue; the E only pushed the broken breadstick’s inner end inside the cover before pulling it back out into its original position (as in experiment 8; Fig. 3b). A novel cue type was used in condition 3: 2-piece uncovered cue; the E temporarily lifted the broken breadstick’s cover so that the subject could see that the breadstick underneath was broken (Fig. 3c and supplementary video 10). A novel setup and a novel cue were used in condition 4: 3-piece middle shown; the E tilted the cover over the broken breadstick so that only the E could see that the breadstick underneath was broken. The E then temporarily removed the middle piece, showed it to the subject and then replaced it in its original position under the cover (Fig. 3d and supplementary video 11). Subjects received 24 trials per condition, and the four conditions were intermixed, which resulted in six sessions of 16 trials that each consisted of four trials of each of the four conditions.

### Data scoring and analysis

As in the experiments above, we used a Binomial test to examine whether subjects’ performance was above chance level. Additionally, we estimated a GLMM including whether an individual chose the intact breadstick (yes, no) as response and species, condition (i.e., cue type: 2-piece slid, 2-piece pushed, 2-piece uncovered, 3-piece middle shown), trial number, and age as fixed factors, and subject was included as random factor. Post-hoc comparisons between the different manipulation conditions were conducted using the package ‘lsmeans’^26^.

### Results

Two-piece slid condition. All 8 subjects chose the intact breadstick more often than by chance: one subject in 100% (Padana; *p* <.001), one in 96% (Tai; *p* <.001), one in 92% (Pini; *p* <.001), two in 88% (Bimbo and Sandra; *p* <.001); two in 83% (Dokana and Azibo; *p* =.002) and one subject in 79% of the test trials (Raja; *p* =.007); Table 1.

Two-piece pushed condition. Five of the eight subjects chose the intact breadstick more often than expected by chance: one subject did so in 100% of the test trials (Padana; *p* <.001), two subjects in 92% (Sandra and Tai, *p* =.006), one in 83% (Bimbo; *p* =.002) and one subject in 75% of the test trials (Azibo; *p* =.023). The remaining three subjects did not pass this test condition (Table 1).

Two-piece uncovered condition. All subjects chose the intact breadstick significantly more often than expected by chance in this control condition in which the broken breadstick’s location was temporarily uncovered. Five subjects chose the intact breadstick in 100% of the test trials (Dokana, Padana, Pini, Sandra, and Tai; *p* <.001) and three subjects did so in 96% of the test trials (Azibo, Bimbo, and Raja; *p* <.001); Table 1.

Three-piece middle shown condition. All subjects chose the intact breadstick significantly above chance levels in the novel transfer test condition: two subjects in 100% (Padana and Tai; *p* <.001), three subjects in 96% (Pini, Raja and Sandra; *p* <.001), one in 92% (Azibo; *p* <.001), one in 79% (Dokana; *p* =.007), and one in 75% of the test trials (Bimbo; *p* =.023); Table 1.

The GLMM revealed that condition (i.e., cue type) had a significant effect on performance but not species (*p* =.563), trial number (*p* =.888), and age (*p* =.979); likelihood ratio test full-null model comparison: *X*^*2*^ = 41.73, *df* = 5, *p* <.001 (Supplementary Table S9a). A post-hoc comparison revealed that individuals performed better in the 3-piece middle shown condition than in the 2-piece pushed condition (*p* =.007) but did not differ in the 2-piece slid and the 2-piece pushed condition (*p* =.110). The**y** also performed better in the 2-piece uncovered condition, in which the breadsticks’ properties were temporarily revealed, than in all other conditions (2-piece slid: *p* =.003; 2-piece pushed: *p* <.001; and 3-piece middle shown: *p* =.028); Supplementary Table S9b.

### Discussion

All subjects were able to correctly choose the intact breadstick when the only information given was to slide the broken breadstick’s end backwards and forwards. This condition had also been used in experiment 3, which only two subjects had passed. Although subjects might have inferred the location of the intact breadstick in the current experiment, based on indirect evidence of the broken breadstick, it is also possible that subjects had just learnt to avoid the broken breadstick. Subjects had been tested with 6 previous experiments in which the broken breadstick was slid back and forth and one of these experiments (7) involved subjects directly observing the broken breadstick’s movement when it was slid backwards and forwards (i.e., without being covered).

Five of the eight subjects were successful when only the broken breadstick was pushed into and pulled out of the cover. The subjects had previously only experienced this condition in experiment 8 in which five subjects had also been successful, but only 3 subjects passed both experiment 8 and experiment 9. In experiment 8, the end of the broken breadstick and the intact breadstick had been pushed and it is possible that subjects were more attracted to choosing the intact breadstick as its outer end moved out of the cover when pushed whereas the outer end of the broken one did not. However, this possibility cannot explain the three subjects’ success in experiment 9 when only the broken breadstick was moved. Although, the results for these three subjects suggest that subjects inferred the breadsticks’ properties it could be argued that they learnt from experiment 8 to avoid the broken breadstick when its inner end was pushed inside. Yet learning cannot explain subjects’ success in the novel conditions.

All subjects successfully chose the intact breadstick when we only uncovered the broken breadstick or just removed the middle piece of the breadstick in the 3-piece condition. It is possible that when subjects saw the broken breadstick when it was uncovered that they just avoided choosing it when it was re-covered without inferring that the intact breadstick had to be under the cover that was not lifted. However, this strategy cannot explain success in the condition in which the middle piece was temporarily removed. In this condition, subjects did not see the broken breadstick being uncovered; they only saw the middle piece being temporarily removed. Subjects would then have to infer that this piece was part of the broken breadstick and subsequently avoid choosing the breadstick from which the middle piece had been taken. Although this condition provides the strongest evidence that subjects in our study were inferring the breadsticks’ properties, other alternative explanations need to be controlled. For example, the E only provided cues about the broken breadstick’s properties and therefore subjects might have been successful by simply avoiding choosing the breadstick that E touched because she never touched the intact breadstick. Similarly, subjects may have simply avoided the breadstick when it was associated with seeing the small middle piece being lifted from the cover. We addressed these possibilities in our final experiment.

## Experiment 10: do orangutans and chimpanzees use non-inferential strategies to succeed in the 3-piece broken breadstick task?

In the previous experiment, it was possible that subjects used non-inferential strategies to avoid the 3-piece broken breadstick. When subjects saw the middle piece being temporarily removed from the cover that the broken breadstick was under, they could have simply used this cue to avoid choosing the broken breadstick. We therefore conducted a new version of the 3-piece broken breadstick task in which this strategy would not result in success. We presented subjects with the 3-piece broken breadstick and the intact breadstick and then each breadstick was hidden under the covers. However, during the hiding process the middle piece was hidden under the same cover as the intact breadstick. When we presented the hidden breadsticks to the subjects, we removed the broken middle piece and showed it to the subjects before replacing it back under the cover with the intact breadstick. We also moved the broken breadstick by pushing its end inside its cover and then pulling it back out. If subjects now used a rule based on avoiding choosing the breadstick associated with the small middle piece, they would be expected to make an incorrect choice by choosing the broken breadstick. However, if subjects were using an inferential strategy, they should reason that when the broken breadstick is moved its pattern of movement could only be physically possible if it was indeed broken. And they should reason that the broken middle piece that they saw being temporarily removed from the cover was not necessarily indicative of a broken breadstick being under the same cover.

Another non-inferential strategy that subjects might have used in the previous experiment was to avoid the 3-piece broken breadstick because it was the only breadstick whose properties were indicated by the experimenter. We now controlled for this strategy by the experimenter providing visual cues on both breadsticks’ properties in the new task.

### Subjects

We tested the 7 of the 8 subjects who had participated in experiment 9.

### Test procedure

The set-up largely corresponded to conditions 2 and 4 from experiment 9. An important difference was that in each test trial, the E hid the broken breadstick’s middle piece under the same cover as the intact breadstick and the broken breadstick’s two ends under the second cover. In each trial, the E then indirectly demonstrated the properties of both breadsticks. When E tilted the cover over the intact breadstick (and the middle piece) so that only the E could see the cover’s contents, the E temporarily lifted the middle piece and then hid it again under the cover. The cue indicating the broken breadstick’s properties consisted in the E pushing the broken breadstick’s inner end inside the cover and pulling it back out into its original position (Fig. 2b and supplementary video 12). Subjects received two daily test sessions of 16 trials each and thus a total of 32 test trials.

### Data scoring and analysis

Data scoring and statistical analyses were the same as in experiment 1. However, in addition to a first GLMM that included all trials (*N* = 32), we estimated a second one that included only *N* = 24 trials in order to better compare performance with the previous experiments, which also consisted of *N* = 24 trials.

### Results

Four of the 7 subjects who participated in this last experiment, chose the intact breadstick significantly more often than expected by chance: one subject chose the intact breadstick in 97% of the test trials (Padana; Binomial test: *p* <.001), one in 84% (Bimbo; Binomial test: *p* <.001), and two subjects did so in 81% (Azibo and Dokana; Binomial tests: *p* <.001) of the test trials (Table 1). All four successful subjects chose the intact breadstick on the first test trial. One of the three unsuccessful subjects only chose the intact breadstick in 31% of the test trials, which was significantly below chance levels (Pini; Binomial test: *p* =.050). Neither species, trial number, nor age affected performance (likelihood ratio test full-null model comparison: *X*^*2*^ = 1.14, *df* = 2, *p* =.567; species: *p* =.325; trial number: *p* =.659; age: *p* =.479; Supplementary Table S10a).

**Fig. 4:**
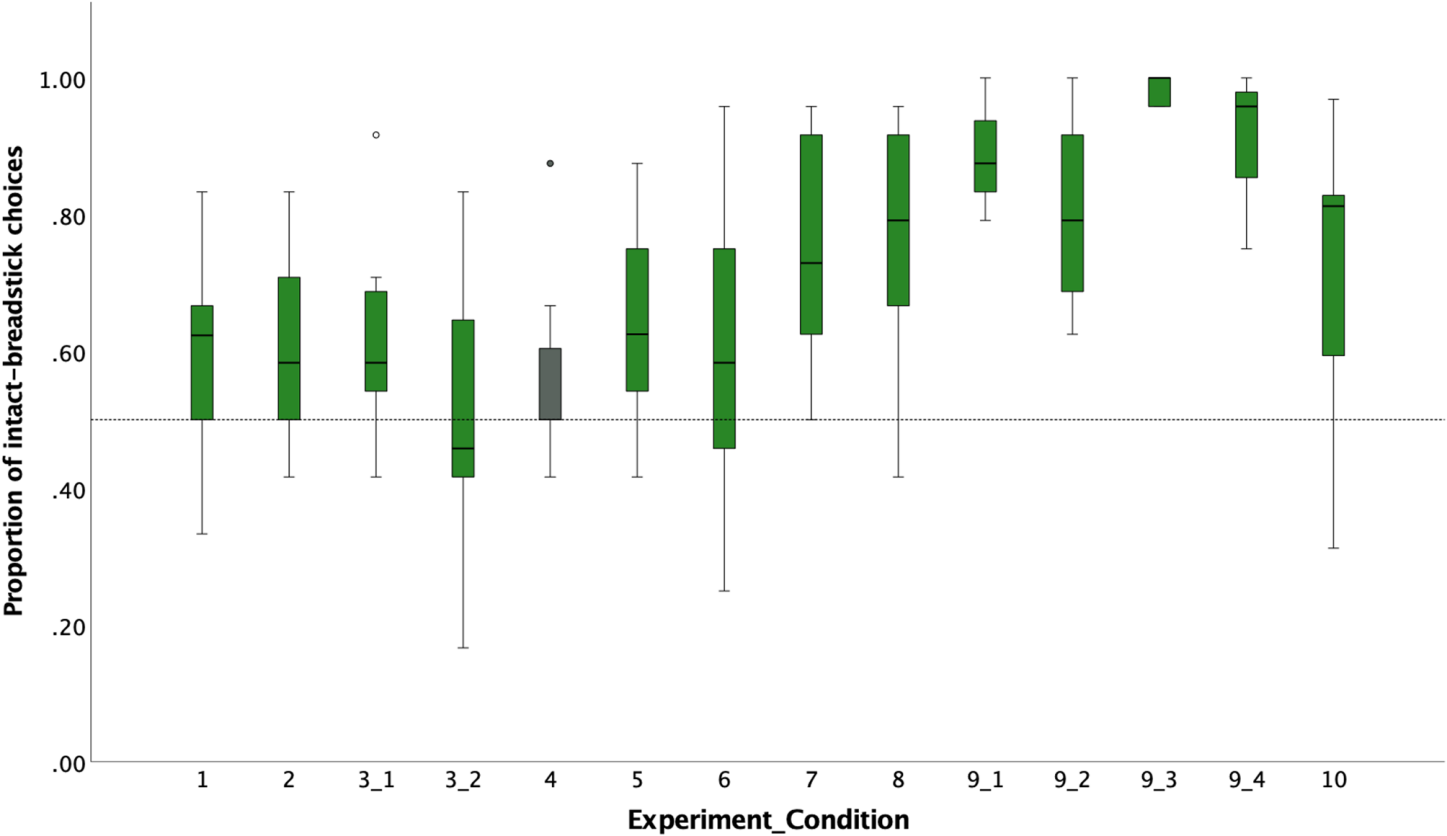
Subjects’ performance in the original experiment (1), the follow-up experiments (2–7) with procedural modifications, the novel breadstick type and cue (9), and the transfer tests (8–10). Procedural modifications: non-delayed choices in exp 2; reduced breadstick movement in exp 3 (condition 1: only intact breadstick slid, condition 2: only broken breadstick slid; outlier: Tai); no movement in exp 4 (control, outlier: Natascha); looks inside covers possible to obtain full visual information about the breadsticks’ hidden properties in exp 5; only intact-breadstick choices rewarded in exp 6; mechanism of breadstick-movement shown prior to the test in exp 7. Transfer tests: novel type of breadstick movement in exp 8: ‘push’ cue; novel ‘2-piece uncovered’ cue in exp 9 (condition 3) and novel 3-piece breadstick type and ‘middle-piece shown‘ cue in exp 9 (condition 4); two conflicting cues in exp 10 (middle piece paired with the intact breadstick). *Error bars: 95% confidence intervals.*

When only analysing the first 24 trials for the purpose of consistency with the previous experiments, these results were confirmed in that the same four individuals were successful and the same three individuals were unsuccessful in choosing the intact breadstick more often than expected by chance. Padana chose the intact breadstick in 96% (Binomial test: *p* <.001), Bimbo in 84% (*p* <.001) and the other three successful subjects in 79% (*p* =.007) of the first 24 test trials whereas Sandra (63%), Tai (50%), and Pini (33%) did not choose the intact breadstick above chance (Table 1). Neither species, trial number, nor age affected performance (likelihood ratio test full-null model comparison: *X*^*2*^ = 3.73, *df* = 2, *p* =.155; species: *p* =.184; trial number: *p* =.153; age: *p* =.235; Supplementary Table S10b and data file 2). For an overview of performance in all experiments see Fig. 4.

### Discussion

We found that four of the 7 subjects, three orangutans and one chimpanzee, reliably chose the intact breadstick. These subjects had been successful in choosing the intact breadstick in the previous experiment (experiment 9) when they were shown the broken middle piece that corresponded to the broken breadstick. However, in experiment 9 subjects might have simply avoided the breadstick that the E touched because she never touched the broken breadstick, or subjects might have used the simple rule: avoid choosing the breadstick under the cover that is associated with the small middle piece. Both strategies would have resulted in subjects choosing the intact breadstick in experiment 9, but they cannot explain the results of the current experiment because the E manipulated both breadsticks, and the middle piece now corresponded to the cover that concealed the intact breadstick. It is therefore possible that subjects were successful in the current experiment by reasoning about the hidden properties of the breadsticks. That is, subjects observed the 3-piece broken breadstick and the intact breadstick before they were hidden inside the covers and then used the inferential cues to reason that when the end of the broken breadstick was pushed into the cover, it must be broken and inferred that the other cover must contain the intact breadstick. This inference would have included reasoning that the broken middle piece being pulled out of the cover containing the intact breadstick had no relevance to the intact breadstick’s properties.

## General discussion

We investigated if orangutans and chimpanzees could reason about the hidden properties of breadsticks. In experiment 1, only one of the 12 subjects showed evidence of this ability indicating that the other subjects were unable to infer the properties of hidden breadsticks. However, an alternative explanation is that factors involved in our experimental procedure hindered the performance of the unsuccessful subjects. We investigated this possibility by conducting several experiments that involved slight procedural modifications of experiment 1. Although we found a modest improvement in the number of subjects passing the task, the experiments only involved slight procedural changes from the original procedure of experiment 1. It could therefore be argued that this improvement was owing to subjects learning to use a non-inferential strategy over the course of the follow-up experiments. Also, in experiment 1 a non-inferential strategy could have been used by the one subject who was successful. It is therefore reasonable to suggest that the results so far showed little evidence that orangutans and chimpanzees could reason about the hidden properties of food. Yet it is also possible that the subjects we tested required some relevant experience before they could make inferences about the hidden properties of breadsticks. Without such experience it is possible that subjects had difficulty reasoning how moving the outer end of each breadstick affected its hidden properties, especially when the broken breadstick was moved. We addressed this possibility in experiment 7 by first giving subjects relevant experience of observing the movement of uncovered breadsticks then retesting subjects with experiment 1. Four of the 8 subjects were successful and two of these had never shown evidence of inferring the properties of the breadsticks in any of the previous 6 experiments. Although relevant experience appeared to help some subjects succeed in experiment 7, these subjects might have selected the intact breadstick in the hidden test trials by simply remembering how each breadstick acted when it was moved in the uncovered experience trials. Subjects were therefore tested with further experiments designed to control for such learnt strategies. Our most interesting finding is that all subjects showed evidence of inferential reasoning when we tested them with a novel broken breadstick consisting of three pieces (two end pieces and a small middle one) that were aligned to resemble the intact breadstick. Once the broken breadstick was hidden under the cover its middle piece could not be seen by the subjects, but during testing it was temporarily removed and shown to the subject who could then use this information to infer that the middle piece belonged to the broken breadstick. All subjects successfully chose the intact breadstick suggesting they were inferring the breadsticks’ properties. It could be argued, however, that subjects were not inferring the properties of the broken breadstick, but they simply avoided choosing the breadstick that was associated with observing a small broken breadstick piece being removed from the cover it was under. Alternatively, subjects might have simply avoided the breadstick the experimenter touched because she only touched the broken one. But these possibilities were ruled out in our final experiment because, unlike the previous experiment, success now depended on selecting the breadstick (intact) that was associated with the broken middle piece being removed and replaced from the intact breadstick’s cover. Also, the experimenter touched both the broken and intact breadstick. This final experiment involved presenting subjects with an intact breadstick and a 3-piece broken breadstick but when the latter was being hidden inside the covers the experimenter surreptitiously placed its broken middle piece under the same cover as the intact breadstick. During the test procedure, the experimenter removed the broken middle piece and showed it to the subject before placing it back under the cover of the intact breadstick. At this point, if subjects were inferring the breadsticks’ properties, they should presume that this breadstick was the 3-piece broken one (when in fact it was the intact one). However, the broken breadstick hidden under the second cover was also manipulated to demonstrate its broken nature, with one broken end being pushed inside the cover and then pulled back out. If subjects had the capacity for inferential reasoning, we expected that when they saw the combination of conflicting cues, that violated their presumed expectations, they could reason that the specific movement of the pushed broken breadstick was only physically possible if it was indeed broken. And although subjects saw a small broken middle piece being lifted from the other cover, we expected them to reason that it was irrelevant to the intact breadstick beneath the same cover. This is because they knew that only one breadstick was intact and one was broken, and they also had already observed the broken breadstick’s movement, which clearly indicated its broken state. Although it could be argued that subjects were successful by again using a non-inferential rule, it is unclear why they would adopt this new rule instead of relying on the one that had already led to success in the previous experiment. Recall that in experiment 9 success could have been based on two rules: (A) avoid choosing the broken breadstick when seeing its small broken piece being removed from the cover, (B) avoid choosing the only breadstick the experimenter touched, which was the broken one. In our final experiment (10) the experimenter touched both breadsticks therefore the non-inferential rules subjects could have used are: (1) avoid choosing the breadstick (broken) that was pushed in and out of the cover, (2) choose the breadstick (intact) when seeing the small broken piece being removed from under its same cover. It seems unlikely that subjects would use rule 2 because success in experiment 9 was dependent on avoiding the small broken piece of breadstick whereas in experiment 10 it was dependent on choosing it. It would be expected that the positive reinforcement of gaining a reward by simply avoiding the small broken piece of breadstick in experiment 9 would have been difficult to immediately reverse when success of gaining a reward in experiment 10 was now dependent on choosing the breadstick associated with the small broken piece of breadstick. For the same reason it seems unlikely that subjects would now switch to using rule 1. Therefore, inferential reasoning may best explain the subjects’ success in the final experiment.

To the best of our knowledge, only one study has employed a paradigm very similar to the one used in our current study to test inferential reasoning in great apes. Mulcahy & Schubiger^17^ tested if orangutans (*Pongo abelii*) could infer the hidden broken or intact properties of wooden sticks that each skewered a food reward. Although subjects initially showed evidence of inferring the properties of the tools, subjects failed control conditions that would have supported inferential reasoning. However, unlike the current study, the orangutans were never given experience of observing the mechanisms involved during the movement of an uncovered broken and intact tool. After such experience with breadsticks, subjects showed evidence of inferential reasoning in experiment 9 and 10. Therefore relevant experience seems to play an important role in the subjects’ understanding of inferential reasoning, which is in line with studies on different aspects of causal reasoning, in which having seen the mechanism also had a beneficial effect on the performance of great apes (e.g^27^). Also, in Mulcahy and Schubiger’s study the task demands may have been too high because subjects were required to reason about two factors simultaneously: the functional properties of the tools and the out-of-reach reward. In our current study we reduced these demands by simply using food that we could alter its intact state to a broken one. This factor could have also contributed to the subjects’ success in our final two experiments.

Some of the conditions in which subjects showed evidence of inferential reasoning in our study involved only providing indirect information about the breadstick that was broken e.g., experiment 9. If subjects were able to reason about the breadsticks’ properties, they could use this information to infer, by exclusion, that the breadstick for which they had not received any indirect information must be the intact one. As discussed earlier, it is suggested that this type of inference by exclusion has been found in great apes (as well as some species of monkeys, domestic mammals and birds) in the A-not-B cup task in which only one reward is hidden under one of two cups. Subjects are shown which cup is empty and they can use this information to infer that the food must be hidden under the cup that was not lifted. However, an alternative non-inferential explanation is that subjects simply avoid the empty cup and therefore choose the correct cup without having any expectation that the reward is hidden inside^7^. This explanation, however, cannot apply to the exclusion method of our study because both covers were never empty. Our method, therefore, is a useful tool to test inferential reasoning by exclusion. Also, our method of using food properties is not as restrictive as using broken and intact tools to investigate inferential reasoning (e.g^17,28^), as this type of method may be more restricted to animal species who use tools. Breadsticks, or other food products with similar properties, can be used to test inferential reasoning in a wide range of non-tool-using animal species.

Overall, our study revealed that orangutans and chimpanzees showed little evidence of inferring the properties of breadsticks when they had not observed the mechanisms involved during the movement of a broken and intact breadstick. However, after subjects had received such experience the majority of subjects showed evidence of inferential reasoning. This was especially the case in our last experiment because the novel nature of the task excluded that success was based on learning, and it controlled for non-inferential strategies that we suggested subjects might use to successfully pass the task.

The main limitation of this study is the small sample size, which necessitates further research with larger samples. This would, for example, help determine whether our findings were influenced by individual or species differences that our study could not adequately capture or control. A larger sample size per species would also enable comparisons between different experimental groups. For instance, comparing subjects with and without prior full visual experience of the mechanism before receiving the original test condition could further clarify the role of task-relevant experience. Also, future research should address if inferential reasoning in the breadstick paradigm extends to other species. Testing birds, for example, would help to clarify if the ability to reason inferentially evolved independently in distantly related taxa. New Caledonian crows (*Corvus moneduloides*) would be ideal candidates owing to their prolific use and manufacture of tools (for a summary and review see^29,30^). Their similar tool-use experience to orangutans and chimpanzees in the current study may provide an advantage to understanding the connective properties of tools. This in turn could help them reason about the hidden broken or intact properties in the food paradigm we used. A species-friendly modified version of the task can be adapted for New Caledonian crows by using strips of intact and nonintact meat. It would also be interesting to establish if inferential reasoning in the breadstick paradigm is possible in species who do not use tools. This would determine the role, if any, tool use plays in non-human animals’ inferential reasoning about the hidden connective properties of breadsticks or similar food products that might be used in future research.

Finally, if future studies establish that our novel task is a reliable and valid test instrument, it has the potential to be suitable for meaningful comparisons of inferential reasoning (and other cognitive abilities) in a wide range of species.

## Supplementary Information

Below is the link to the electronic supplementary material.

Supplementary Material 1

Supplementary Material 2

Supplementary Material 3

Supplementary Material 4

Supplementary Material 5

Supplementary Material 6

Supplementary Material 7

Supplementary Material 8

Supplementary Material 9

Supplementary Material 10

Supplementary Material 11

Supplementary Material 12

Supplementary Material 13

Supplementary Material 14

Supplementary Material 15

Supplementary Material 16

## Data Availability

The raw data are provided within the manuscript in Table 1 and in the supplementary data files 1 and 2. The R-script used for the GLMMs is available in the supplementary code script.

## References

[1] Premack, D. & Premack, A. J. Levels of causal Understanding in chimpanzees and children. *Cognition* **50**(1–3), 347–362. 10.1016/0010-0277(94)90035-3 (1994). 8039368 10.1016/0010-0277(94)90035-3

[2] Grether, W. F. & Maslow, A. H. An experimental study of insight in monkeys. *J. Comp. Psychol.* **24**, 127–134. 10.1037/h0057666 (1937).

[3] Call, J. Inferences about the location of food in the great apes (*Pan paniscus, Pan troglodytes, Gorilla gorilla, and Pongo pygmaeus)*. *J. Comp. Psychol.* **118**(2), 232–241. 10.1037/0735-7036.118.2.232 (2004). 15250810 10.1037/0735-7036.118.2.232

[4] Hill, A., Collier-Baker, E. & Suddendorf, T. Inferential reasoning by exclusion in great apes, lesser apes, and spider monkeys. *J. Comp. Psychol.* **125**(1), 91–103. 10.1037/a0020867 (2011). 21341913 10.1037/a0020867

[5] Engelmann, J. M. et al. Do chimpanzees reason logically? *Child. Dev.* **00**, 1–15. 10.1111/cdev.13861 (2022). 10.1111/cdev.13861 36259153

[6] Sabbatini, G. & Visalberghi, E. Inferences about the location of food in capuchin monkeys (*Cebus apella*) in two sensory modalities. *J. Comp. Psychol.* **122**(2), 156–166. 10.1037/0735-7036.122.2.156 (2008). 18489231 10.1037/0735-7036.122.2.156

[7] Paukner, A., Huntsberry, M. E. & Suomi, S. J. Tufted capuchin monkeys (*Cebus apella*) spontaneously use visual but not acoustic information to find hidden food items. *J. Comp. Psychol.* **123**(1), 26–33. 10.1037/a0013128 (2009). 19236142 10.1037/a0013128 PMC2648131

[8] Schmitt, V. & Fischer, J. Inferential reasoning and modality dependent discrimination learning in Olive baboons (*Papio Hamadryas anubis*). *J. Comp. Psychol.* **123**(3), 316. 10.1037/a0016218 (2009). 19685974 10.1037/a0016218

[9] Marsh, H. L., Vining, A. Q., Levendoski, E. K. & Judge, P. G. Inference by exclusion in lion- tailed macaques (*Macaca silenus*), a Hamadryas baboon (*Papio Hamadryas*), capuchins (*Sapajus apella*), and squirrel monkeys (*Saimiri sciureus)*. *J. Comp. Psychol.* **129**, 256–267. 10.1037/a0039316 (2015). 26010194 10.1037/a0039316

[10] Erdőhegyi, Á., Topál, J., Virányi, Z. & Miklósi, Á. Dog-logic: Inferential reasoning in a two-way choice task and its restricted use. *Anim. Behav.* **74**(4), 725–737. 10.1016/j.anbehav.2007.03.004 (2007).

[11] Nawroth, C. & von Borell, E. Domestic pigs’ (*Sus scrofa domestica*) use of direct and indirect visual and auditory cues in an object choice task. *Anim. Cogn.* **18**(3), 757–766. 10.1007/s10071-015-0842-8 (2015). 25650328 10.1007/s10071-015-0842-8

[12] Nawroth, C., von Borell, E. & Langbein, J. Exclusion performance in Dwarf goats (*Capra Aegagrus hircus*) and sheep (*Ovis orientalis aries*). *PloS One*. **9**(4), e93534. 10.1371/journal.pone.0093534 (2014). 24695781 10.1371/journal.pone.0093534 PMC3973590

[13] Mikolasch, S., Kotrschal, K. & Schloegl, C. African grey parrots (*Psittacus erithacus*) use inference by exclusion in to find hidden food. *Biol. Lett.* **7**(6), 875–877. 10.1098/rsbl.2011.0500 (2011). 21697165 10.1098/rsbl.2011.0500 PMC3210682

[14] Pepperberg, I. M., Koepke, A., Livingston, P., Girard, M. & Hartsfield, L. A. Reasoning by inference: further studies on exclusion in grey parrots (*Psittacus erithacus*). *J. Comp. Psychol.* **127**(3), 272. 10.1037/a0031641 (2013). 23421751 10.1037/a0031641

[15] Paukner, A., Anderson, J. R. & Fujita, K. Redundant food searches by capuchin monkeys (*Cebus apella*): a failure of metacognition? *Anim. Cogn.* **9**(2), 110–117. 10.1007/s10071-005-0007-2 (2006). 16184375 10.1007/s10071-005-0007-2

[16] Call, J. The avoid the empty cup hypothesis does not explain great apes’ (*Gorilla gorilla, Pan paniscus, Pan troglodytes, Pongo abelii*) responses in two three-cup one-item inference by exclusion tasks. *J. Comp. Psychol.* **136**(3), 172–188. 10.1037/com0000321 (2022). 35771524 10.1037/com0000321

[17] Mulcahy, N. J. & Schubiger, M. N. Can orangutans (*Pongo abelii*) infer tool functionality? *Anim. Cogn.* **17**, 657–669. 10.1007/s10071-013-0697-9 (2014). 24132413 10.1007/s10071-013-0697-9

[18] Mulcahy, N. J., Schubiger, M. N. & Suddendorf, T. Orangutans (*Pongo Pygmaeus and Pongo abelii*) understand connectivity in the skewered grape tool task. *J. Comp. Psychol.* **127**(1), 109–113. 10.1037/a0028621 (2013). 22686164 10.1037/a0028621

[19] Jacobs, I. & Osvath, M. The String-Pulling paradigm in comparative psychology. *J. Comp. Psychol.* **129**(2), 89–122. 10.1037/a0038746 (2015). 25984937 10.1037/a0038746

[20] Mulcahy, N. J. Orangutans (*Pongo abelii*) seek information about tool functionality in a metacognition tubes task. *J. Comp. Psychol.* **130**, 391–399. 10.1037/com0000046 (2016). 27841455 10.1037/com0000046

[21] Bohn, M., Allritz, M., Call, J. & Voelter, C. J. Information seeking about tool properties in great apes. *Sci. Rep.* **7**, 10923. 10.1038/s41598-017-11400-z (2017). 28883523 10.1038/s41598-017-11400-z PMC5589724

[22] Landis, J. R. & Koch, G. G. The measurement of observer agreement for categorical data. *Biometrics* **33**, 159–174. 10.2307/2529310 (1977). 843571

[23] Schielzeth, H. & Forstmeier, W. Conclusions beyond support: overconfident estimates in mixed models. *Behav. Ecol.* **20**, 416–420. 10.1093/beheco/arn145 (2009). 19461866 10.1093/beheco/arn145 PMC2657178

[24] Forstmeier, W. & Schielzeth, H. Cryptic multiple hypotheses testing in linear models: overestimated effect sizes and the winner’s curse. *Behav. Ecol. Sociobiol.* **65**, 47–55. 10.1007/s00265-010-1038-5 (2011). 21297852 10.1007/s00265-010-1038-5 PMC3015194

[25] Call, J. & Carpenter, M. Do apes and children know what they have seen? *Anim. Cogn.* **3**, 207–220. 10.1007/s100710100078 (2001).

[26] Lenth, R., VLeast-Squares & Means The R package Lsmeans. *J. Stat. Softw.* **69**(1), 1–33. 10.18637/jss.v069.i01 (2016).

[27] Völter, C. & Call, J. Problem solving in great apes (*Pan paniscus, Pan troglodytes, Gorilla gorilla, and Pongo abelii*): the effect of visual feedback. *Anim. Cogn.* **15**, 923–936. 10.1007/s10071-012-0519-5 (2012). 22644115 10.1007/s10071-012-0519-5

[28] Seed, A., Seddon, E., Greene, B. & Call, J. Chimpanzee ‘folk physics’: bringing failures into focus. *Philos. Trans. R Soc. B : Biol. Sci.* **367**(1603), 2743–2752. 10.1098/rstb.2012.0222 (2012). 10.1098/rstb.2012.0222 PMC3427557 22927573

[29] Klump, B. C. Of crows and tools. *Science* **366**(6468), 965. 10.1126/science.aaz7775 (2019). 31753993 10.1126/science.aaz7775

[30] Rutz, C. & J Clair, J. The evolutionary origins and ecological context of tool use in new Caledonian crows. *Behav. Processes*. **89**, 153–165. 10.1016/j.beproc.2011.11.005 (2012). 22209954 10.1016/j.beproc.2011.11.005

