# Supplementary material for: Orangutans and chimpanzees show evidence of inferring when a hidden breadstick is intact or broken
Source: Sci Rep. 2026 Feb 27;16:11305. doi: 10.1038/s41598-026-38796-x (PMC13049003; doi:10.1038/s41598-026-38796-x)
Supplement: Supplementary file 3 — Supplementary Material 3 [file 41598_2026_38796_MOESM3_ESM.pdf]

Supplementary information

Orangutans and chimpanzees show evidence of inferring when a hidden breadstick is intact or broken

M. N. Schubiger*[1,2,3,4], C. Fichtel[1,2], & N. J. Mulcahy[4]

Affiliations:

[1]Behavioural Ecology & Sociobiology Unit, German Primate Center, Göttingen, Germany

[2]Leibniz ScienceCampus 'Primate Cognition', Göttingen, Germany

[3] Department of Evolutionary Anthropology, University of Zurich, Zürich, Switzerland

[4]World Ape Fund, London, United Kingdom

Corresponding author: Michèle N. Schubiger; mnschubiger@protonmail.com

Supplementary Tables - Results of the GLMMs

The response variable was success (i.e., the proportion of intact-breadstick-choices) and subject was included as a random effect in all models. Species, trial number, age, and test condition (where applicable) were included as fixed effects. Significance level: $P < .05$.

Table S1 No effect of species, trial number, or age on success in experiment 1.

Fixed effects	*Estimate*	*SE*	*z*	*P*
Intercept	-0.04	0.52	-0.07	0.944
Species	0.29	0.35	0.82	0.410
Trial	-0.13	0.17	-0.76	0.445
Age	0.15	0.17	0.88	0.379

Table S2 No effect of species, trial number, or age on success in experiment 2.

Fixed effects	*Estimate*	*SE*	*z*	*P*
Intercept	0.70	0.53	1.33	0.185
Species	-0.17	0.35	-0.48	0.633
Trial	-0.07	0.17	-0.40	0.688
Age	-0.32	0.18	-1.79	0.074

Table S3 Effect of condition on success in experiment 3 Subjects performed better in condition 1 (*intact slid*) than in condition 2 (*broken slid*).

Fixed effects	*Estimate*	*SE*	*z*	*P*
Intercept	0.32	0.45	0.72	0.469
Species	0.11	0.28	0.38	0.704
Condition	0.45	0.18	-2.49	**0.013**
Trial	-0.11	0.09	-1.24	0.214
Age	0.03	0.14	0.23	0.816

Table S4 Effect of trial number on success in experiment 4 Negative effect of trial number on success in this control condition, in which no visual cues were provided (i.e., none of the breadsticks were moved).

Fixed effects	*Estimate*	*SE*	*z*	*p*
Intercept	0.83	0.42	1.98	0.048
Species	-0.41	0.27	-1.50	0.133
Trial	-0.27	0.13	-2.08	**0.038**
Age	0.22	0.14	1.63	0.103

Table S5 Effect of looks and trial on success in experiment 5 Positive effect of number of looks (i.e., obtain full visual information after having watched the two indirect visual cues) and negative effect of trial number.

Fixed effects	*Estimate*	*SE*	*z*	*p*
Intercept	-0.01	0.52	-0.02	0.982
Species	0.49	0.32	1.54	0.125
Looks	0.75	0.22	3.41	**0.001**
Trial	-0.46	0.16	-2.91	**0.004**
Age	-0.28	0.15	-1.86	0.063

Table S6 No effect of species, trial number, or age success in experiment 6.

Fixed effects	*Estimate*	*SE*	*z*	*p*
Intercept	0.01	0.79	0.01	0.993
Species	0.46	0.49	0.93	0.350
Trial	-0.23	0.15	-1.50	0.133
Age	-0.16	0.24	-0.67	0.504

Table S7 No effect of species, trial number or age on success in experiment 7.

Fixed effects	*Estimate*	*SE*	*z*	*p*
Intercept	1.24	1.43	0.87	0.384
Species	0.01	0.85	0.01	0.992
Trial	-0.03	0.17	-0.15	0.878
Age	0.05	0.40	0.11	0.911

Table S8 Effect of species on success in experiment 8 Novel version of the inference task: *pushed* cue. (i.e., the intact and broken breadstick were temporarily pushed inside the covers). Orangutans performed marginally better than chimpanzees.

Fixed effects	*Estimate*	*SE*	*z*	*p*
Intercept	-1.00	1.21	-0.83	0.407
Species	1.47	0.74	1.99	**0.046**
Trial	0.22	0.18	1.19	0.232
Age	-0.36	0.36	-0.99	0.322

Table S9a Effect of condition (i.e., visual cue type) on performance in experiment 9 The subjects' performance in condition 1 (2-piece *slid*) differed from their performance in conditions 2 (2-piece *pushed*) and 3 (2-piece *uncovered*) but not condition 4 (3-piece *middle shown*).

Fixed effects	*Estimate*	*SE*	*z*	*p*
Intercept	3.00	1.33	2.62	0.024
Species	-0.45	0.79	-0.58	0.563
Condition 2	-0.67	0.30	-2.25	**0.024**
Condition 3	2.13	0.62	3.44	**0.001**
Condition 4	0.37	0.35	1.06	0.291
z.trial	0.02	0.13	0.14	0.889
z.age	-0.01	0.37	-0.03	0.980

Table S9b Posthoc tests Subjects performed better in condition 4 (3-piece *middle shown*) than in condition 2 (2-piece *pushed*) and better in condition 3 (2-piece *uncovered*) than in all other conditions.

Pair-wise differences of condition/cue type	*Estimate*	*SE*	*z*	*p*
Cond 1 – Cond 2	0.67	0.30	2.25	0.110
Cond 1 – Cond 3	-2.13	0.62	-3.44	**0.003**
Cond 1 – Cond 4	-0.37	0.35	-1.06	0.716
Cond 2 – Cond 3	-2.81	0.61	-4.60	**< 0.001**
Cond 2 – Cond 4	-1.04	0.32	-3.23	**0.007**
Cond 3 – Cond 4	1.77	0.63	2.78	**0.028**

Table S10a No effects of species, trial number, or age on success in experiment 10.

Fixed effects	*Estimate*	*SE*	*z*	*p*
Intercept	-0.63	1.77	-0.36	0.721
Species	1.09	1.11	0.98	0.325
Trial	-0.07	0.16	-0.44	0.659
Age	-0.38	0.53	-0.71	0.479

Table S10b No effects of species, trial number, or age on success in the first 24 trials experiment 10.

Fixed effects	*Estimate*	*SE*	*z*	*p*
Intercept	-1.19	1.61	-0.74	0.462
Species	1.36	1.02	1.33	0.184
Trial	-0.26	0.18	-1.43	0.153
Age	-0.60	0.51	-1.19	0.235
