# Supplementary material for: Orangutans and chimpanzees show evidence of inferring when a hidden breadstick is intact or broken
Source: Sci Rep. 2026 Feb 27;16:11305. doi: 10.1038/s41598-026-38796-x (PMC13049003; doi:10.1038/s41598-026-38796-x)
Supplement: Supplementary file 5 — Supplementary Material 5 [file 41598_2026_38796_MOESM5_ESM.pdf]

Supplementary information

Orangutans and chimpanzees show evidence of inferring when a hidden breadstick is intact or broken

M. N. Schubiger*[1,2,3,4], C. Fichtel[1,2], & N. J. Mulcahy[4]

Affiliations:

[1]Behavioural Ecology & Sociobiology Unit, German Primate Center, Göttingen, Germany

[2]Leibniz ScienceCampus 'Primate Cognition', Göttingen, Germany

[3] Department of Evolutionary Anthropology, University of Zurich, Zürich, Switzerland

[4]World Ape Fund, London, United Kingdom

Corresponding author: Michèle N. Schubiger; mnschubiger@protonmail.com

R-Code Script

```
rm(list=ls())

ls()


library(lme4)


xdata=read.table("~/inference_data.csv", header=TRUE, sep=";", dec=",",stringsAsFactors=T)

str(xdata)

table(xdata$subject)

table(xdata$subject, xdata$species)


### Experiment 1: Can orangutans and chimpanzees infer food functionality after observing indirect information about the functional properties of breadsticks?####

exp.1<-subset(xdata, xdata$experiment=="Exp_1")

nrow(exp.1)

hist(exp.1$age)
```

transformation of data

exp.1$z.age=as.vector(scale(exp.1$age))

exp.1$z.trial_S=as.vector(scale(exp.1$trial_S))

fit.1<-glmer(success ~ species + z.trial_S + z.age + (1|subject), data=exp.1, family=binomial)

summary(fit.1)

fit.10<-glmer(success ~ z.age + (1|subject), data=exp.1, family=binomial)

anova(fit.10, fit.1, test="Chisq")

Experiment 2: Can orangutans and chimpanzees infer food functionality after observing indirect information about the functional properties of breadsticks when they can immediately make their choice?

exp.2<-subset(xdata, xdata$experiment=="Exp_2")

nrow(exp.2)

transformation of data

exp.2$z.age=as.vector(scale(exp.2$age))

exp.2$z.trial_S=as.vector(scale(exp.2$trial_S))

fit.2<-glmer(success ~ species + z.trial_S + z.age + (1|subject), data=exp.2, family=binomial)

summary(fit.2)

fit.20<-glmer(success ~ z.age + (1|subject), data=exp.2, family=binomial)

anova(fit.20, fit.2, test="Chisq")

Experiment 3: Can orangutans and chimpanzees infer food functionality after seeing indirect information about the functional properties of only one of two breadsticks?

exp.3<-subset(xdata, xdata$experiment=="Exp_3")

nrow(exp.3)

transformation of data

exp.3$z.age=as.vector(scale(exp.3$age))

exp.3$z.trial_S=as.vector(scale(exp.3$trial_S))

```
fit.3<-glmer(success ~ species + condition_cue_type + z.trial_S + z.age + (1|subject), data=exp.3, family=binomial)

summary(fit.3)

fit.30<-glmer(success ~ z.age + (1|subject), data=exp.3, family=binomial)

anova(fit.30, fit.3, test="Chisq")


### Experiment 4: Can orangutans and chimpanzees visually distinguish the breadsticks in the absence of indirect visual information about food functionality? ####

exp.4<-subset(xdata, xdata$experiment=="Exp_4")

nrow(exp.4)


### transformation of data ####

exp.4$z.age=as.vector(scale(exp.4$age))

exp.4$z.trial_S=as.vector(scale(exp.4$trial_S))


fit.4<-glmer(success ~ species + z.trial_S + z.age + (1|subject), data=exp.4, family=binomial)

summary(fit.4)


fit.40<-glmer(success ~ z.age + (1|subject), data=exp.4, family=binomial)

anova(fit.40, fit.4, test="Chisq")


### Experiment 5: Can orangutans and chimpanzees infer food functionality based solely on indirect visual information or do they seek direct visual information?  ####

exp.5<-subset(xdata, xdata$experiment=="Exp_5")

nrow(exp.5)


### transformation data ####

exp.5$z.age=as.vector(scale(exp.5$age))

exp.5$z.trial_S=as.vector(scale(exp.5$trial_S))

exp.5$z.looks=as.vector(scale(exp.5$looks))

fit.5<-glmer(success ~ species + z.looks + z.trial_S + z.age + (1+z.looks|subject), data=exp.5, family=binomial)

summary(fit.5)


fit.50<-glmer(success ~ z.age + (1+z.looks|subject), data=exp.5, family=binomial)
```

```r
anova(fit.50, fit.5, test="Chisq")

### Experiment 6: Does the subjects’ performance of inferring food functionality improve when only one of two possible breadstick choices is rewarded? ####

exp.6<-subset(xdata, xdata$experiment=="Exp_6")

nrow(exp.6)

### transformation of data ####

exp.6$z.age=as.vector(scale(exp.6$age))

exp.6$z.trial_S=as.vector(scale(exp.6$trial_S))

fit.6<-glmer(success ~ species + z.trial_S + z.age + (1|subject), data=exp.6, family=binomial)

summary(fit.6)

fit.60<-glmer(success ~ z.age + (1|subject), data=exp.6, family=binomial)

anova(fit.60, fit.6, test="Chisq")

### Experiment 7: Does the subjects' performance improve after experiencing the functional mechanism of each breadstick when they are moved? ####

exp.7<-subset(xdata, xdata$experiment=="Exp_7")

nrow(exp.7)

### transformation data ####

exp.7$z.age=as.vector(scale(exp.7$age))

exp.7$z.trial_S=as.vector(scale(exp.7$trial_S))

fit.7<-glmer(success ~ species + z.trial_S + z.age + (1|subject), data=exp.7, family=binomial)

summary(fit.7)

fit.70<-glmer(success ~ z.age + (1|subject), data=exp.7, family=binomial)

anova(fit.70, fit.7, test="Chisq")

### Experiment 8: Can orangutans and chimpanzees infer food functionality in a different novel task? ####

exp.8<-subset(xdata, xdata$experiment=="Exp_8")

nrow(exp.8)
```

transformation of data

exp.8$z.age=as.vector(scale(exp.8$age))

exp.8$z.trial_S=as.vector(scale(exp.8$trial_S))

fit.8<-glmer(success ~ species + z.trial_S + z.age + (1|subject), data=exp.8, family=binomial)

summary(fit.8)

fit.80<-glmer(success ~ z.age + (1|subject), data=exp.8, family=binomial)

anova(fit.80, fit.8, test="Chisq")

table(exp.8$species, exp.8$success)

Experiment 9: Can orangutans and chimpanzees use indirect information to infer food functionality when 1) only the broken breadstick is pushed, and 2) when the indirect visual cues do not involve any asymmetry in breadstick movement?

exp.9<-subset(xdata, xdata$experiment=="Exp_9")

nrow(exp.9)

transformation of data

exp.9$z.age=as.vector(scale(exp.9$age))

exp.9$z.trial_S=as.vector(scale(exp.9$trial_S))

fit.9<-glmer(success ~ species + condition_cue_type + z.trial_S + z.age + (1|subject), data=exp.9, family=binomial)

summary(fit.9)

fit.90<-glmer(success ~ z.age + (1|subject), data=exp.9, family=binomial)

anova(fit.90, fit.9, test="Chisq")

post-hoc comparisons condition_cue_type

library(emmeans)

lsmeans(fit.9,list(pairwise~condition_cue_type),adjust="tukey")

Experiment 10: Do orangutans and chimpanzees use non-inferential strategies to succeed in the 3-piece broken breadstick task?

```
exp.10<-subset(xdata, xdata$experiment=="Exp_10")

nrow(exp.10)


### transformation of data ####

exp.10$z.age=as.vector(scale(exp.10$age))

exp.10$z.trial_S=as.vector(scale(exp.10$trial_S))

fit.10<-glmer(success ~ species + z.trial_S + z.age + (1|subject), data=exp.10, family=binomial)

summary(fit.10)


fit.100<-glmer(success ~ z.age + (1|subject), data=exp.10, family=binomial)

anova(fit.100, fit.10, test="Chisq")


##subset for only first 24 trial_Ss##

xdata_b=read.table("~/Documents/AAABBBCCC/_Aktuelles/_to_review/Michele/January/inference_data_exp
_b.csv", header=TRUE, sep=";", dec=",",stringsAsFactors=T)


### transformation of data ####

xdata_b$z.age=as.vector(scale(xdata_b$age))

xdata_b$z.trial_S=as.vector(scale(xdata_b$trial_S))


fit.10b<-glmer(success ~ species + z.trial_S + z.age + (1|subject), data=xdata_b, family=binomial)

summary(fit.10)


fit.10b0<-glmer(success ~ z.age + (1|subject), data=xdata_b, family=binomial)

anova(fit.10b0, fit.10b, test="Chisq")
```
